# *CIC/ATXN1*‐rearranged tumors in the central nervous system are mainly represented by sarcomas: A comprehensive clinicopathological and epigenetic series

**DOI:** 10.1111/bpa.13303

**Published:** 2024-10-23

**Authors:** Arnault Tauziède‐Espariat, Azadeh Ebrahimi, Nathalie Boddaert, Torsten Pietsch, Wieslawa Grajkowska, Tobias Blau, Arend Koch, Philipp Sievers, Delphine Guillemot, Gaëlle Pierron, Emmanuelle Uro‐Coste, Yvan Nicaise, Aurore Siegfried, Adam Gilles, Franck Bielle, Karima Mokhtari, Dominique Cazals‐Hatem, Gueorgui Iakovlev, Benoît Lhermitte, Natacha Entz‐Werle, Marie Csanyi, Claude‐Alain Maurage, Victor Legrand, Jean Boutonnat, Catherine Godfraind, Anne McLeer, Lauren Hasty, Alice Métais, Oumaima Aboubakr, Thomas Blauwblomme, Kévin Beccaria, Volodia Dangouloff‐Ros, Pascale Varlet, The RENOCLIP‐LOC

**Affiliations:** ^1^ Department of Neuropathology, GHU Paris‐Psychiatrie et Neurosciences Sainte‐Anne Hospital Paris France; ^2^ INSERM U1266, IMABrain Institute of Psychiatry and Neuroscience of Paris (IPNP), Université Paris Cité Paris France; ^3^ Department of Neuropathology, DGNN Brain Tumor Reference Center University of Bonn Bonn Germany; ^4^ Pediatric Radiology Department Hôpital Necker Enfants Malades, AP‐HP Paris France; ^5^ UMR 1163, Institut Imagine and INSERM U1299 Université Paris Cité Paris France; ^6^ Department of Pathology The Children's Memorial Health Institute Warsaw Poland; ^7^ Institute for Neuropathology University of Duisburg‐Essen Essen Germany; ^8^ Department of Neuropathology Charité – Universitätsmedizin Berlin, Corporate Member of Freie Universität Berlin and Humboldt‐Universität zu Berlin Berlin Germany; ^9^ Department of Neuropathology, Institute of Pathology University Hospital Heidelberg Heidelberg Germany; ^10^ Clinical Cooperation Unit Neuropathology, German Consortium for Translational Cancer Research (DKTK) German Cancer Research Center DKFZ) Heidelberg Germany; ^11^ INSERMU830 Institut Curie Research Center, Paris‐Sciences‐Lettres Paris France; ^12^ Laboratory of Somatic Genetics Institut Curie Hospital Paris France; ^13^ Department of Pathology Toulouse University Hospital Toulouse France; ^14^ INSERM U1037 Cancer Research Center of Toulouse (CRCT) Toulouse France; ^15^ Université Paul Sabatier, Toulouse III Toulouse France; ^16^ Department of Neuroradiology Toulouse University Hospital Toulouse France; ^17^ Department of Neuropathology Pitié‐Salpêtrière Hospital, AP‐HP Paris Paris France; ^18^ Department of Pathology APHP University Hospital Beaujon Clichy France; ^19^ Department of Neurosurgery APHP University Hospital Beaujon Clichy France; ^20^ Department of Pathology Strasbourg Hospital Strasbourg France; ^21^ Department of Pediatric Oncology Strasbourg Hospital Strasbourg France; ^22^ Department of Biopathology Lille University Hospital Lille France; ^23^ Department of Neurosurgery Lille University Hospital Lille France; ^24^ Department of Pathology Grenoble University Hospital La Tronche France; ^25^ Neuropathology Unit, UMR 1071 Clermont‐Ferrand University Hospital and Université Clermont‐Auvergne Clermont‐Ferrand France; ^26^ Molecular Pathology Unit, Department of Pathology Grenoble Alpes University, Grenoble University Hospital Grenoble France; ^27^ Department of Pediatric Neurosurgery Necker Hospital, APHP, Université Paris Descartes, Sorbonne Paris Cité Paris France

**Keywords:** ATXN1, CIC, DNA methylation profile, glioneuronal, LEUTX, sarcoma

## Abstract

*CIC* fusions have been described in two different central nervous system (CNS) tumor entities. On one hand, fusions of *CIC* or *ATXN1* genes belonging to the same complex of transcriptional repressors, were reported in the *CIC‐*rearranged, sarcoma (SARC*‐CIC*). The diagnosis of this tumor type, which was recently added to the World Health Organization (WHO) Classification of CNS tumors, is difficult mainly because the data concerning its histopathology (as compared to its soft tissue counterpart), immunoprofile, and clinical as well as radiological characteristics are scarce in the literature. On the other hand, a recent study, based on DNA‐methylation profiling, has identified a novel high‐grade neuroepithelial tumor characterized by recurrent *CIC* fusions (HGNET‐*CIC*). The aim of this multicentric study was to characterize a cohort of 15 primary CNS tumors harboring a *CIC* or *ATXN1* fusion in terms of clinical, radiological, histopathological, immunophenotypical, and epigenetic characteristics. According to the integrated diagnoses, 14/15 tumors corresponded to SARC*‐CIC,* and only one to HGNET‐*CIC*. The tumors showed similar clinical (mainly pediatric), radiological (mostly supratentorial, cystic, and contrast enhancing), immunophenotypical (common expression of glioneuronal markers), and genetic (similar spectrum of fusions) profiles but their histopathological appearance was clearly distinct. Moreover, we found a novel fusion transcript (*CIC::EWSR1*) in a SARC‐*CIC.* Most DNA methylation profiles using the Heidelberg Brain Tumor Classifier (v12.8) annotated the samples to the methylation class “SARC‐*CIC*” (9/14 tumors with available data). By using uniform manifold approximation and projection analysis, four other samples were classified as SARC‐*CIC* and another clustered within the methylation class of HGNET‐*CIC*. Our findings confirm that CNS *CIC*‐fused tumors do not represent a single molecular tumor entity. Further analyses are needed to characterize HGNET‐*CIC* in more detail. These results may help to refine the essential diagnostic criteria for SARC‐*CIC* and their terminology (with a suggested consensual name of sarcoma, *CIC/ATXN1‐*complex rearranged).

## Graphical abstract

Main characteristics of sarcomas, CIC‐rearranged and HGNET, CIC‐fused.

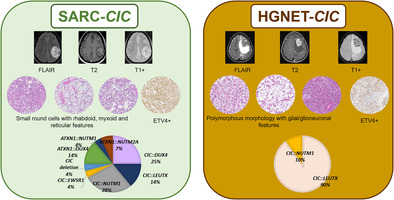

## 1 INTRODUCTION

The latest World Health Organization's (WHO) Classification of Central Nervous System (CNS) Tumors, published in 2021, considerably modified the chapter on mesenchymal, non‐meningothelial tumors [1, 2]. It included novel tumor types, such as the *CIC‐*rearranged sarcoma (SARC‐*CIC*) which was already known in the soft tissue [3, 4]. Based on their similar histopathology, the literature seems to argue in favor of the same tumor type occurring in two different anatomical locations. Thus, this diagnosis constitutes a novel challenge for neuropathologists who are unfamiliar with this rare and newly defined mesenchymal neoplasm. Within the CNS, the clinicopathological and radiological data for SARC*‐CIC* remains scarce in the literature, limited to case reports or small series (*n* = 31) [5, 6, 7, 8, 9, 10, 11, 12, 13, 14, 15, 16, 17, 18]. Moreover, *CIC‐*fused CNS tumors seem to present particularities compared to their soft tissue counterparts in terms of phenotype (a glioneuronal differentiation/immunoprofile has been reported in CNS tumors) [8, 14, 15, 18] and genotype. Whereas a *CIC::DUX4* fusion is encountered in 95% of SARC*‐CIC* of the soft tissues, the molecular spectrum of CNS tumors seems to be broader with different fusion partners (*NUTM1, LEUTX, DUX4*) which may or may not include the gene *CIC* [5, 6, 7, 8, 9, 10, 11, 12, 13, 14, 15, 16, 17, 18]. *ATXN1* fusions are exceptional in soft tissues [18, 19]. Furthermore, to make things more complex, an epigenetically defined high‐grade neuroepithelial tumor with *CIC* fusion (HGNET‐*CIC*) has been recently identified, harboring a recurrent *CIC::LEUTX* fusion, [20]. Very few clinicopathological data are available for this recent entity. As a result, the essential diagnostic criteria for SARC‐*CIC* defined by the current WHO classification ([1] evidence of a *CIC* gene fusion and [2] predominant round cell phenotype; and [3] mild nuclear pleomorphism; and [4] variable admixture of epithelioid and/or spindle cells; and [5] variably myxoid stroma; and [6] variable CD99 and frequent ETV4 and WT1 expression) are no longer up‐to‐date and need to be reevaluated [1]. The aims of our work were to extensively characterize a cohort of CNS tumors from the *CIC‐*fused spectrum in terms of clinical data, imaging, histopathology and molecular biology, and to try to specify precise diagnostic criteria.

## 2 METHODS

### 2.1 Sample collection

The tumor samples and patients' retrospective clinical data were provided by the consultation archive database (1982–2023) from the neuropathology department at GHU Paris‐Psychiatry and Neurosciences Sainte‐Anne Hospital and by French expert centers within the RENOCLIP‐LOC network (*n* = 11). Four additional patients were added from the Institute of Neuropathology, University of Bonn Medical Center in Germany. Case selection was based on the primary CNS tumors with a *CIC* rearrangement evidenced by Fluorescent in situ hybridization (FISH) analysis and/or with a *CIC* or *ATXN1* fusion. The tissue analyses were performed in accordance with local ethics regulations. Four cases were previously reported in the literature (#11 in [20], #12 in [15], #14 in [11], and #15 in [6]).

### 2.2 Clinical and radiological data

The following clinical data were acquired for each patient when possible: sex, age at imaging diagnosis, age at pathological diagnosis, past medical history, clinical presentation, quality of resection, radiotherapy, chemotherapy, event‐free survival (EFS) and overall survival (OS). When available, a central radiological review by experienced neuroradiologists (VDR, and NB) was performed. The following features were evaluated: location, size, diffusion signal and apparent coefficient diffusion (ADC), contrast enhancement, presence of cysts, necrosis, edema, and perfusion parameters.

### 2.3 Histopathological review and immunohistochemical analyses

A representative paraffin block was selected for each tumor. Hematoxylin‐Phloxin‐Saffron (HPS)‐stained slides for all samples underwent central review by an experienced neuropathologist (ATE). Histopathological patterns were labeled as oligodendroglial‐like, astrocytic‐like, ependymoma‐like (with pseudorosettes), small round cells, epithelioid, reticular, rhabdoid, spindle cells, and microcystic. Necrosis, microvascular proliferation, Rosenthal fibers, eosinophilic granular bodies, ganglion cells, neuropil islands, microcalcifications, siderophages (deposition of iron pigment in macrophages suggesting a past hemorrhage), perivascular lymphocytic infiltrates, fibrotic or myxoid changes were noted as present or absent. The tumoral pattern was assessed as circumscribed, diffuse, or both, based on the histopathology for instance the presence of entrapped neurons in the tumor and by using neurofilament staining. The mitotic index was monitored using 10 high‐power fields (HPF; corresponding to 3.2 mm^2^) in a hot‐spot area and was counted by two neuropathologists. Reticulin staining was also performed.

Unstained 3‐μm‐thick slides of formalin‐fixed, paraffin‐embedded tissues were obtained and submitted for immunostaining. The following primary antibodies were used: ETV4 (1:100, clone PEA3, Santa Cruz Biotechnology; Dallas, USA), NUT (1:100, clone C52B1, Sigma‐Aldrich, Saint‐Louis, USA), Glial Fibrillary Acidic Protein (GFAP) (1:200, clone 6F2, Dako, Glostrup, Denmark), OLIG2 (1:3000, clone C‐17, Santa Cruz Biotechnology, Dallas, USA), vimentin (1:800, clone V9, Dako, Glostrup, Denmark), epithelial membrane antigen (1:200, clone GM008, Dako, Glostrup, Denmark), CKAE1AE3 (1:800, clone AE1AE3, Dako, Glostrup, Denmark), CD99 (1:10, clone 12E7, Dako, Glostrup, Denmark), S100 (1:200, clone 6F2, Dako, Glostrup, Denmark), SOX10 (1:200, clone IHC010, Diagomics, Blagnac, France), Neurofilament Protein (1:100, clone 2F 11, Dako, Glostrup, Denmark), NeuN (1:1000, clone A60, Sigma‐Aldrich, Saint‐Louis, USA), CD56 (pre‐diluted, clone 123C3, Dako, Glostrup, Denmark), synaptophysin (1:150, clone DAK‐SYNAP, Dako, Glostrup, Denmark), alpha‐smooth muscle actin (1:6000, clone S100, Dako, Glostrup, Denmark), desmin (1:200, clone D33, Dako, Glostrup, Denmark), CD34 (1:40, clone Qbend10, Dako, Glostrup, Denmark), INI1 (BAF47) (1:50, clone 25/BAF 47, BD Biosciences, Franklin Lakes, USA), BRG1 (1:50, clone EPR3912, Abcam, Cambridge, United Kingdom), and Ki‐67 (1:200, clone MIB‐1, Dako, Glostrup, Denmark). External positive and negative controls were used for all antibodies. MIB‐1 labeling index was estimated in a hot‐spot area by two neuropathologists.

### 2.4 DNA methylation array processing and copy number profiling

Genomic DNA was extracted from fresh‐frozen or formalin‐fixed and paraffin‐embedded (FFPE) tissue samples. DNA methylation profiling of all samples was performed using the Infinium MethylationEPIC (850 k) BeadChip (Illumina, San Diego, CA, USA) or the Infinium HumanMethylation450 (450 k) BeadChip array (Illumina) as previously described [21]. All computational analyses were performed in R version 3.3.1 (R Development Core Team, 2016; https://www.R-project.org). Copy‐number variation analyses from 450 k and EPIC methylation array data were performed using the Conumee Bioconductor package version 1.12.0. Raw signal intensities were obtained from IDAT‐files using the Minfi Bioconductor package version 1.21.4 [21]. Illumina EPIC samples and 450 k samples were merged into a combined data set by selecting the intersection of probes present on both arrays (combineArrays function, minfi). Each sample was individually normalized by performing a background correction (shifting of the 5% percentile of negative control probe intensities to 0) and a dye‐bias correction (scaling of the mean of normalization control probe intensities to 10,000) for both color channels. Subsequently, a correction for the type of material tissue (FFPE/frozen) and array type (450 k/EPIC) was performed by fitting univariable, linear models to the log2‐transformed intensity values (removeBatchEffect function, limma package version 3.30.11). The methylated and unmethylated signals were corrected individually. Beta‐values were calculated from the retransformed intensities using an offset of 100 (as recommended by Illumina). All samples were checked for duplicates by pairwise correlation of the genotyping probes on the 450 k/850 k array. To perform unsupervised non‐linear dimension reduction, the remaining probes after standard filtering [21] were used to calculate the 1‐variance weighted Pearson correlation between samples. The following non‐default parameters were applied: theta = 0, pca = F, max_iter = 20,000 perplexity = 10. Dimensionality reduction was then performed using the uniform manifold approximation and projection (UMAP) method (uwot R package) with the following non‐default parameters: n_neighbors = 10, spread = 2, min‐dist = 0.2.

## 3 RESULTS

### 3.1 Clinical and radiological characteristics

**FIGURE 1:**
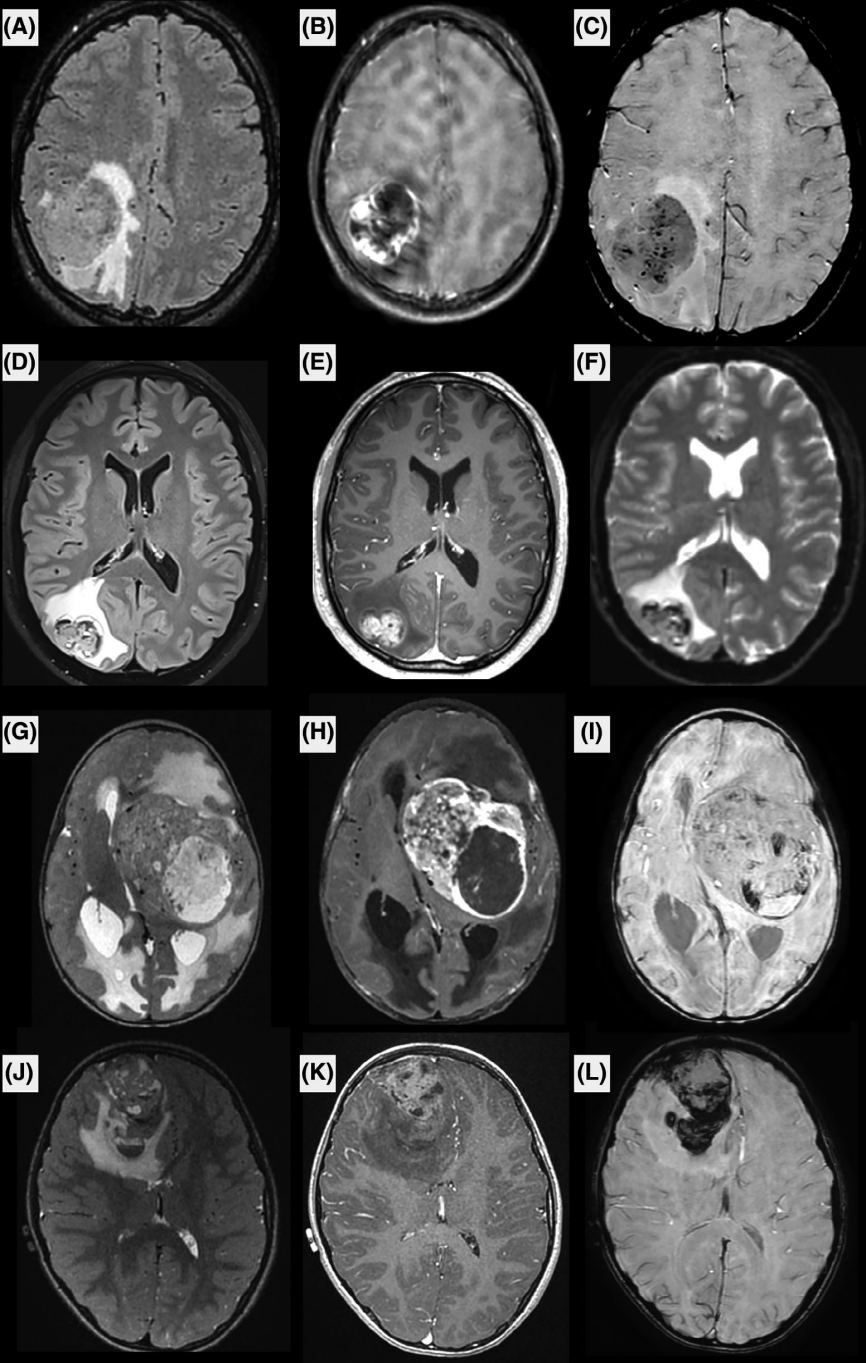
Radiological features of *CIC*‐rearranged sarcomas. Axial FLAIR (A, D), T2‐weighted (G, J), postcontrast T1‐weighted (B, E, H, K), and Susceptibility‐weighted (C, I, L) or echo‐planar (F) images of cases #1 (A, B, C), #3 (D, E, F), #5 (G, H, I) and #6 (J, K, L). The tumors were located supratentorially, and had partially cystic content, intense contrast enhancement, intra‐tumoral hemorrhage, and large peritumoral edema. Most of the tumors had pachymeningeal contact, which is clearly seen in patient #6.

**FIGURE 2:**
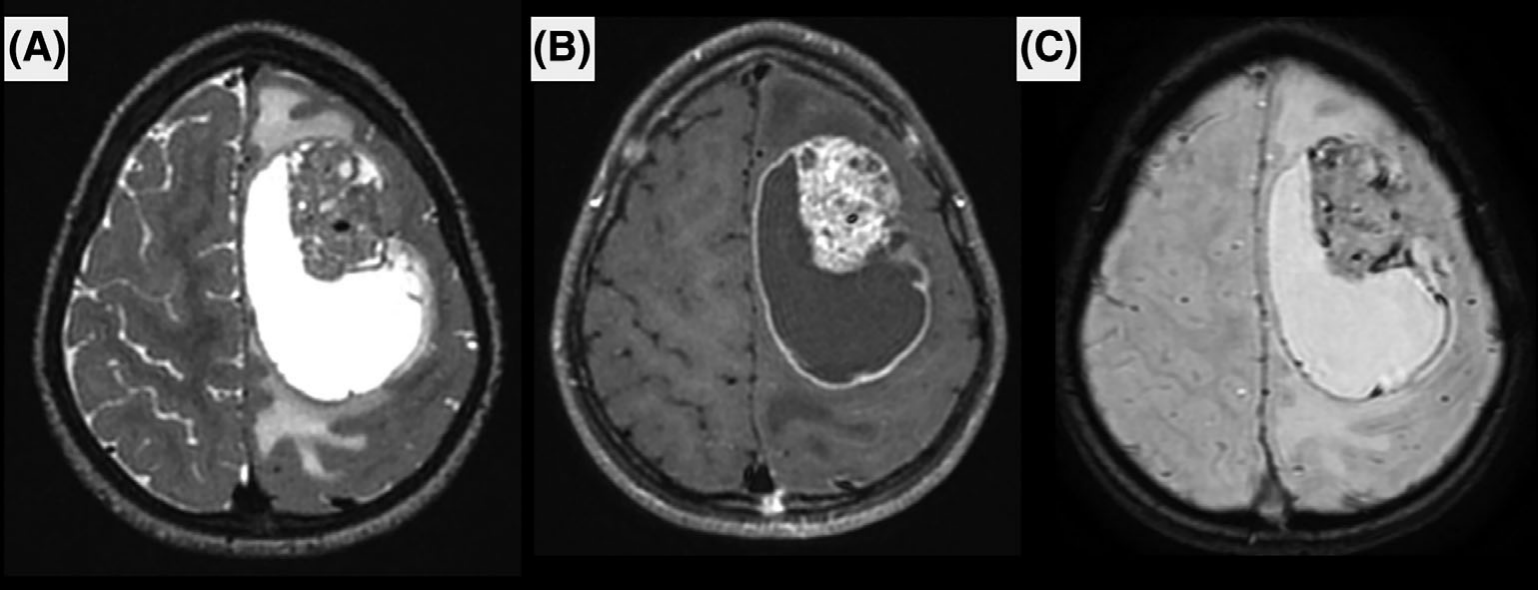
Radiological features of the *CIC*‐rearranged high‐grade neuroepithelial tumor. Images show a large left fontal tumor, with cystic content and peritumoral edema on T2‐weighted images (A), intense contrast enhancement (B) and intra‐tumoral microbleeds (C).

The patients' clinical and neuroradiological data are detailed in Table S1 and Figures 1 and 2 and Supplementary Figure 1. The median age of presentation in our cohort was 9.5 years (ranging from 0 to 40). Ten patients (67%) were children. The female‐to‐male ratio was 2.0 (10 females and 5 males). The tumors were mostly supratentorial (13/15) but two were cerebellar. Data from treatment and follow‐up was available for 9/15 patients. A gross total resection was achieved in 6/9 patients with no residual disease as verified by the postoperative central neuroradiological review. Two patients underwent focal radiation therapy combined with chemotherapy, one patient received focal radiation therapy alone and two patients received only adjuvant chemotherapy. Four patients were free of disease at 1–60 months. Eight patients presented a local recurrence after the initial resection (mean EFS, 2 months; median EFS, 1 month), and six of them died rapidly after the recurrence (median OS, 17.5 months; mean OS, 18.5 months for the whole cohort).

MRI was available for 9/15 patients, including pre‐ and post‐contrast images, diffusion and perfusion imaging was available for seven. The images showed common features among the nine patients. All were large tumors (median largest diameter 52 mm, range [32–81]) with marked peritumoral edema (>1 cm) and intense contrast enhancement. Hemorrhage was seen in all cases, either as multiple microhemorrhages or gross tumor hemorrhage. Peripheral cysts with enhancing walls were seen in 8/9 patients, while microcysts within the tumor tissue were seen in 6/9 patients. Diffusion was restricted in 6/9, intermediate in 2/9 and uninterpretable for the remaining patient (median minimum relative ADC 0.98, IQR [0.82–1.14]). Perfusion was high in most cases using arterial spin labeling or dynamic susceptibility contrasting, except for one patient. All tumors were intra‐axial, with 4/9 showing a large section of pachymeningeal contact, 3/9 showing limited pachymeningeal contact, and 1/9 cases showed only slight leptomeningeal contact. The last tumor had contact with the choroid plexus of the lateral ventricle without peripheral meningeal contact. None of the patients had brain metastases or spinal metastases when spinal MRI was available at diagnosis (3/9).

### 3.2 Histopathological and immunohistochemical characterization

**FIGURE 3:**
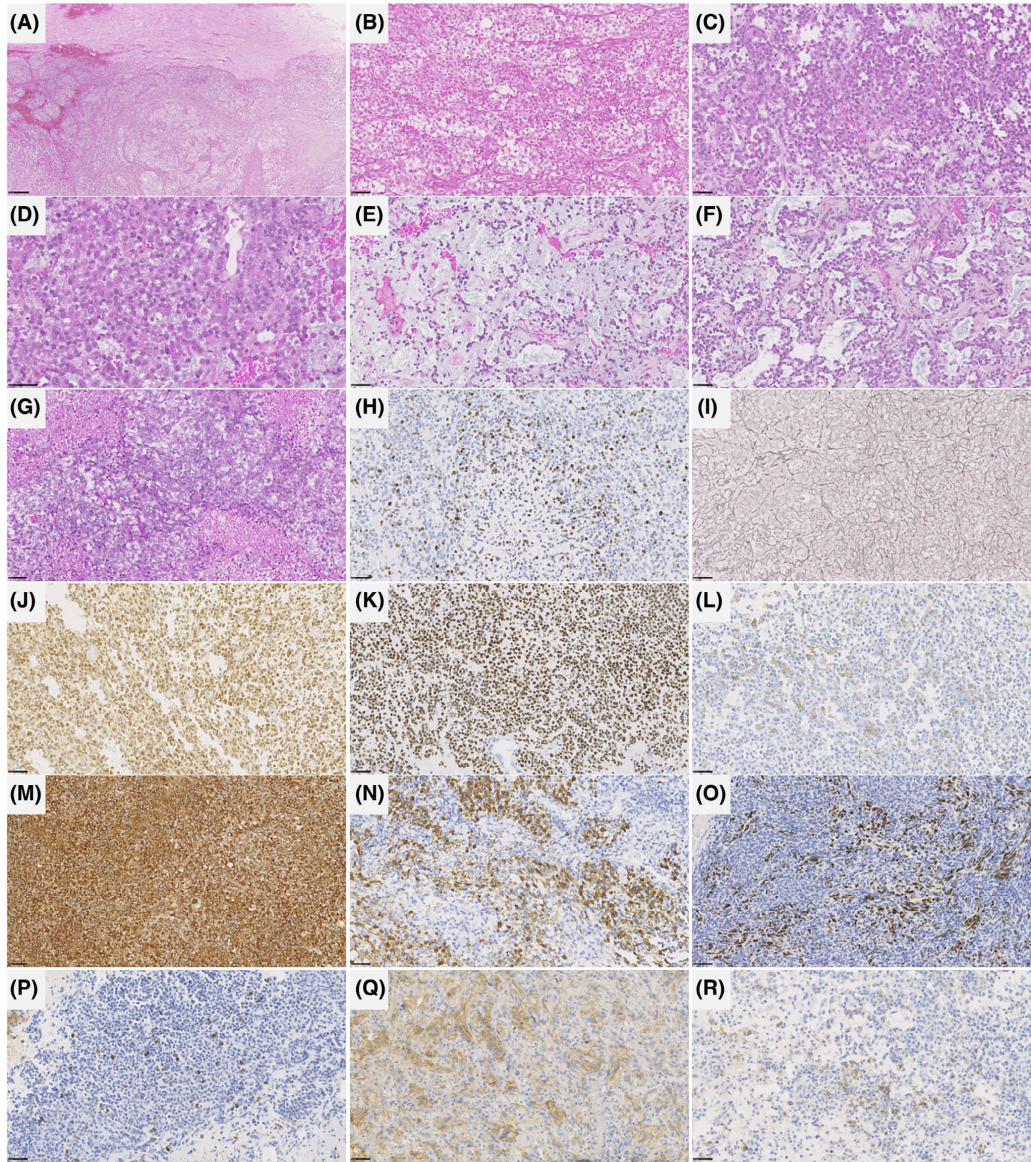
Histopathological features of *CIC*‐rearranged sarcomas. (A) Well‐defined delineation of the tumor from the brain parenchyma (HPS, magnification ×80). (B) Sheets of cells with a lobular appearance and epithelioid differentiation (HPS, magnification ×400). (C) Sheets of small round cells (HPS, magnification ×400). (D) Rhabdoid features (HPS, magnification ×600). (E) Myxoid modifications (HPS, magnification ×400). (F) A reticular pattern was present in a subset of cases (HPS, magnification ×400). (G) Areas of necrosis (HPS, magnification ×400). (H) High MIB1 labeling index (magnification ×400). (I) Reticulin deposition (magnification ×400). (J) Diffuse immunopositivity for ETV4 (magnification ×400). (K) Diffuse immunoreactivity for NUT in a tumor with a *CIC::NUTM1* fusion (magnification ×400). (L) A focal membranous staining for CD99 (magnification ×400). (M) Diffuse expression of vimentin (magnification ×400). (N) Expression of GFAP by a subset of tumor cells (magnification ×400). (o) Expression of OLIG2 in some tumor cells (magnification ×400). (P) Expression of neurofilament by some tumor cells (magnification ×400). (Q) Expression of CD56 (magnification ×400). (R) Expression of synaptophysin by a subset of tumor cells (magnification ×400). Black scale bars represent 250 μm (A) and 50 μm (B–R). HPS, Hematoxylin Phloxin Saffron.

**FIGURE 4:**
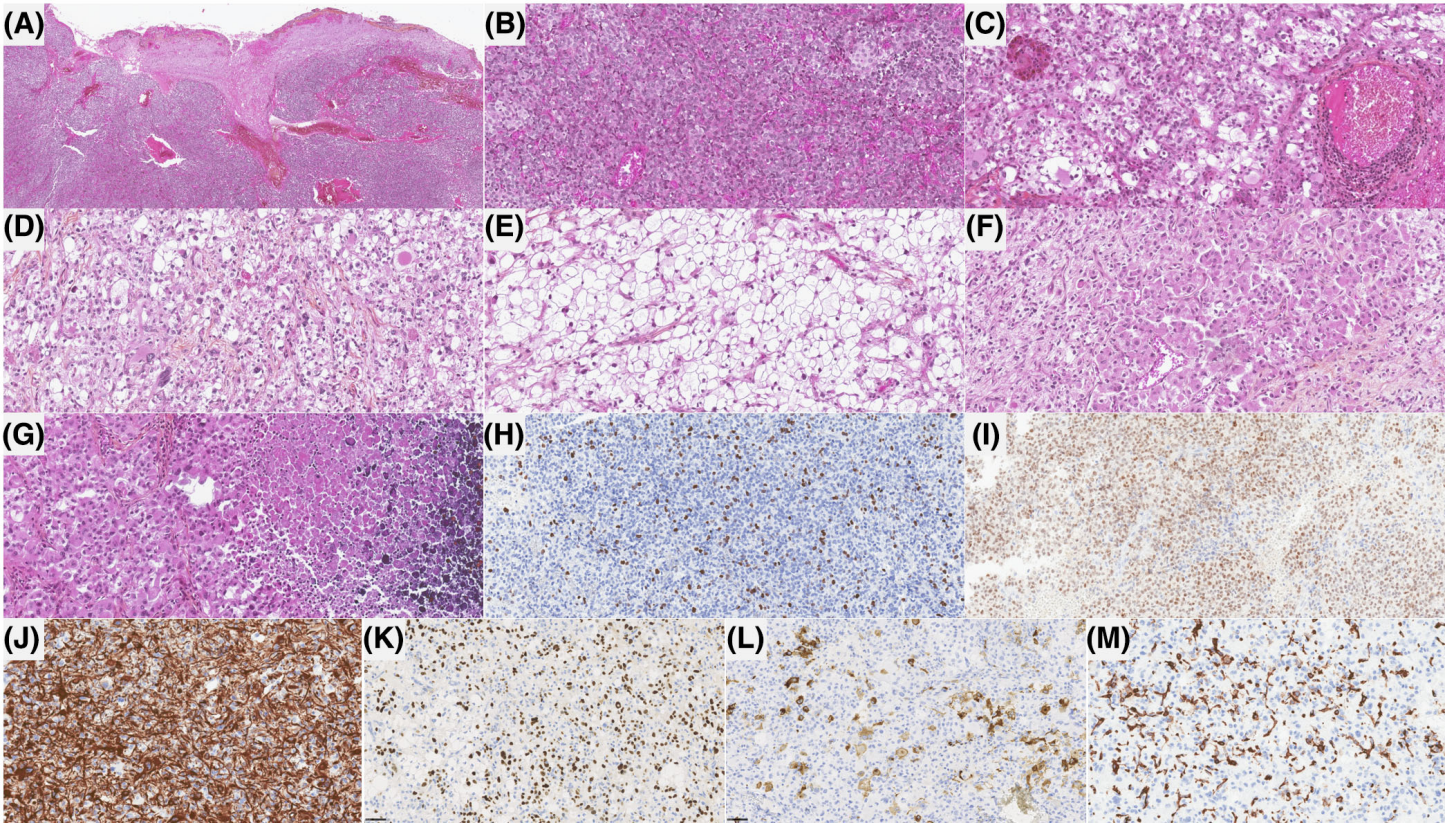
Histopathological features of the *CIC*‐rearranged high‐grade neuroepithelial tumor. (A) Well‐defined delineation of the tumor from the brain parenchyma (HPS, magnification ×80). (B) Sheets of small round cells (HPS, magnification ×400). (C, D) Glioneuronal features with perivascular lymphocytic infiltrates and eosinophilic granular bodies (HPS, magnification ×400). (E) A microcystic pattern (HPS, magnification ×400). (F) A glial gemistocytic pattern (HPS, magnification ×400). (G) Area of necrosis (HPS, magnification ×400). (H) High MIB1 labeling index (magnification ×400). (I) Diffuse immunopositivity for ETV4 (magnification ×400). (J) Diffuse expression of GFAP (magnification ×400). (K) Expression of OLIG2 in some tumor cells (magnification ×400). (L) Expression of synaptophysin by some tumor cells (magnification ×400). (M) An extravascular cellular expression of CD34 (magnification ×400). HPS, Hematoxylin Phloxin Saffron.

The histopathological data of the patients are detailed in Table S1 and illustrated in Figures 3 and 4. All tumors were well‐circumscribed from the brain or cerebellar parenchyma. Fourteen tumors presented similar compositions of dense sheets of tumor cells, with a variable amount of septa (often abundant) on silver impregnation, giving a vague lobular appearance. Using reticulin staining, there was no single cell ensheathment. A small round cells component was constantly observed and represented the predominant pattern in tumors admixed with spindle cells (7/14), a rhabdoid (5/14), or an epithelioid component (2/14). No ependymal (particularly pseudorosettes), astrocytic, oligodendroglial or glioneuronal features were present in those cases. No eosinophilic granular bodies, neuropil islands or perivascular inflammatory infiltrates were evident. Microcystic (5/14) and reticular (3/14) architecture were present. The stroma frequently presented myxoid changes (9/14), and one displayed a chondroid matrix. Hemorrhagic modifications were constantly present but hemosiderin depositions or siderophages were absent. The vascularization was well‐developed with thin‐walled branching vessels and focal microvascular proliferation in only two tumors.

Necrosis was obvious in 10/14 cases and mitoses were frequent (11–31 per 1.6 mm^2^, with a mean of 22 mitoses). Ki‐67 labeling activity was high, ranging from 15% to 80% (median: 30%). By immunohistochemistry, all tumors diffusely expressed vimentin. Epithelial (CKAE1/AE3 in one case, and EMA in four tumors), muscular (smooth muscle actin and desmin in two cases), neuronal (10/14), and glial (GFAP in 7/14 tumors, and OLIG2 in 5/14 cases) markers were variably expressed. INI1 and BRG1 immunoexpressions were constantly retained. A membranous staining for CD99 was present in 11/13 tested samples, mainly focal. ETV4 was constantly expressed whereas NUT immunopositivity was observed in five tumors.

One case (#14) was clearly distinct, having a polymorphous morphology composed of a predominant glioneuronal pattern with eosinophilic granular bodies, perivascular inflammatory infiltrates, CD34 extravascular cellular staining, GFAP, OLIG2 and synaptophysin expression in a large part of the tumor cells, admixed with clear cells, small round cells, and focal epithelioid cells. By immunohistochemistry, tumor cells also expressed vimentin, and ETV4. However, there was no immunoexpression for epithelial and myogenic markers. CD99 was focally expressed in this tumor. The silver impregnation showed a single cell ensheathment. Necrosis was obvious and mitoses were frequent (21 per 1.6 mm^2^). Ki‐67 labeling activity was high (40%).

### 3.3 Genetic and epigenetic features, and integrated diagnoses

Alterations of the *CIC* gene were present in 12/15 cases, including: *CIC::DUX4* (*n* = 5), *CIC::NUTM1* (*n* = 4), *CIC::LEUTX* (*n* = 2), and *CIC::EWSR1* (*n* = 1). The three remaining tumors presented an *ATXN1::DUX4* (*n* = 2), and an *ATXN1::NUTM1* (*n* = 1) fusion. First, DNA‐methylation profiles were generated from the 14 tumor samples that had sufficient DNA quality. Using DNA methylation‐based classification, nine of the tumors were classified as SARC*‐CIC* (calibrated scores for DNA MC ≥0.9), and seven of them were shown to harbor a *CIC* fusion. Four other samples were classified as SARC*‐CIC* but with a low calibrated score (<0.9), and the last case was not classified. Next, we performed an UMAP analysis, including the recently described MC of HGNET‐*CIC* [20], of the whole cohort to better classify the tumors with low calibrated scores (<0.9). Thirteen tumors clustered with SARC*‐CIC* reference tumors. Because they presented similar histopathological characteristics, the integrated diagnosis of each was SARC‐*CIC*. The remaining case (#14) grouped within the class of HGNET‐*CIC* reference tumors (Supplementary Figure 2). As it was morphologically different from the other tumors in our series, we considered an integrated diagnosis of HGNET‐*CIC*.

No recurrent copy number variations were observed in the two subgroups (Supplementary Figure 3).

## 4 DISCUSSION

**FIGURE 5:**
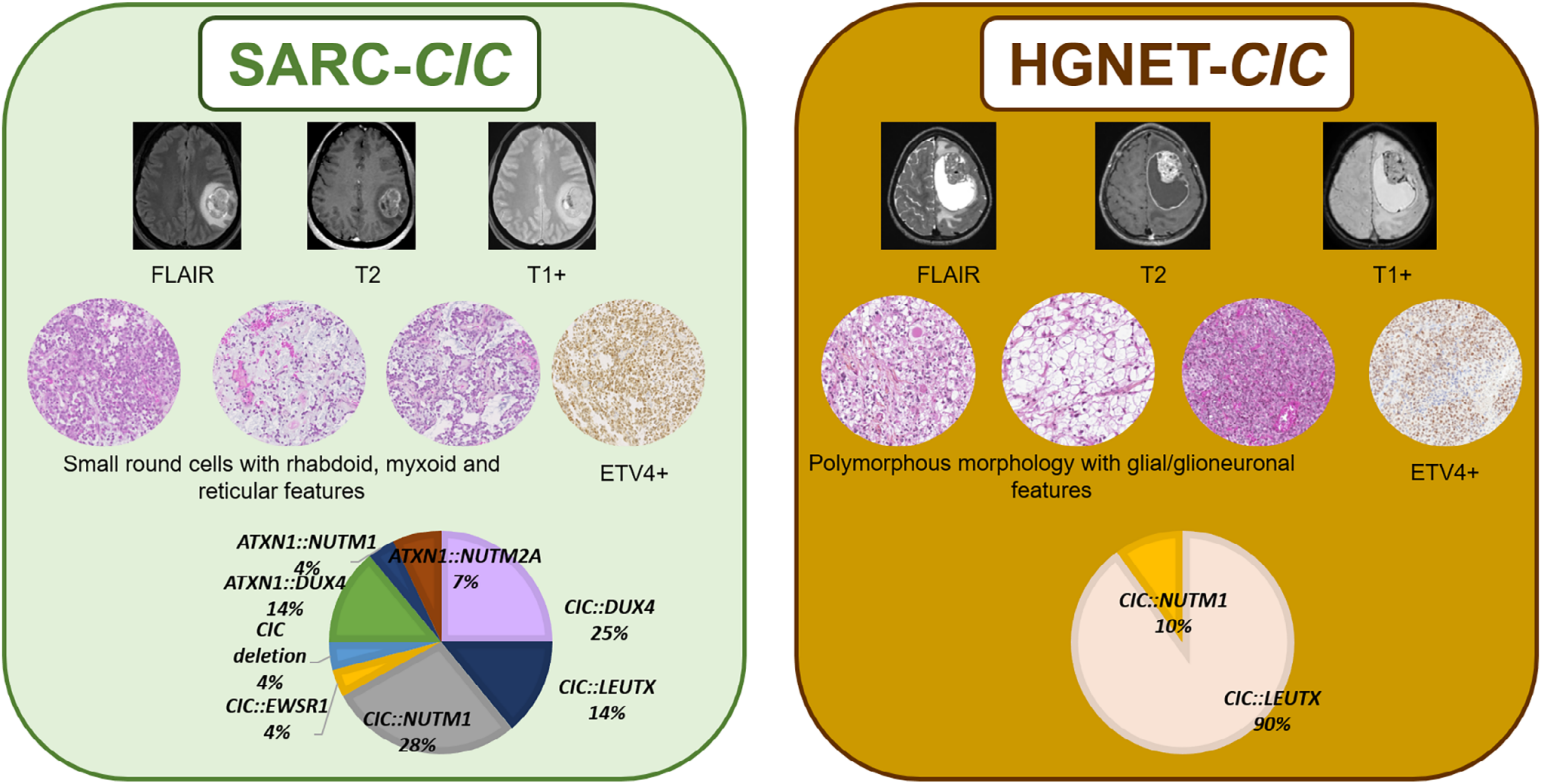
Summary of *CIC*‐rearranged sarcomas and *CIC*‐rearranged high‐grade neuroepithelial tumors main characteristics extracted from the literature and the current series. Both SARC‐*CIC* and HGNET‐*CIC* were large tumors with marked peritumoral edema and intense contrast enhancement. Hemorrhage was seen in all tumors. SARC‐*CIC* present densely cellular sheets of small round cells with rhabdoid differentiation, myxoid modifications and reticular pattern whereas HGNET‐*CIC* showed obvious signs of neuronal differentiation (eosinophilic granular bodies, perivascular lymphocytic infiltrates and ganglion cells), and other components (clear cells and small round cells). The two entities share the expression of ETV4. SARC‐*CIC* present a wide variety of alterations (mostly fusions) implicating *CIC* or *ATXN1* genes whereas HGNET‐*CIC* are associated to date with *CIC::LEUTX* or *CIC::NUTM1* fusions. HGNET‐*CIC*, high‐grade neuroepithelial tumor, *CIC‐*fused; SARC‐*CIC*, *CIC*‐rearranged, sarcoma.

In the CNS, two different entities are now known to harbor recurrent *CIC/ATXN1* fusions: the newly WHO CNS 2021 defined SARC*‐CIC* and the recently described HGNET‐*CIC* [5, 20] (Figure 5). In the current work, our comprehensive detailed study includes clinical, radiological, histopathological and genetic data from a series of CNS tumors with *CIC* spectrum fusions, mainly represented by SARC*‐CIC*. They presented frequently as a small round cell morphology admixed with variable epithelioid, rhabdoid or spindle cells. As previously reported, SARC*‐CIC* constantly expressed ETV4 [22] and may express the protein NUT for those harboring a *NUTM1* fusion [5]. Surprisingly, these sarcomas may express markers from different lineages (epithelial, myogenic, glial and neuronal), which explains why some of them were initially diagnosed in the literature as high‐grade gliomas or embryonal tumors [5, 7, 8, 15]. The expression of neuronal and epithelial markers has also been reported in SARC‐*CIC* of soft tissues [23, 24, 25, 26, 27], but to our knowledge, no data are available about glial expression in extra‐CNS tumors. As was observed in the current series, SARC*‐CIC* mainly affect children and young adults (median age: 8 years), with 70% of patients being found in the pediatric age group [5, 6, 7, 8, 9, 10, 11, 12, 13, 14, 15, 16, 17, 18]. There is no gender predisposition (sex ratio male/female of 1.1) in the literature [5, 6, 7, 8, 9, 10, 11, 12, 13, 14, 15, 16, 17, 18]. Most SARC*‐CIC* of the CNS occur in supratentorial sites (85% of reported tumors) [37–46] whereas spinal and posterior fossa (the two first examples are in the current series) presentation accounts for 10% and 5% of reported cases, respectively [5, 6, 7, 8, 9, 10, 11, 12, 13, 14, 15, 16, 17, 18]. For the first time, we showed that SARC*‐CIC* may present recurrent imaging features. These are characterized by large intra‐axial tumors with frequent meningeal contact, marked peritumoral edema, intense contrast enhancement, hemorrhagic foci, diffusion restriction, and high perfusion. Because of the age of onset, morphology and pluriphenotypic immunoprofile, the main differential diagnosis for SARC‐*CIC* is atypical teratoid and rhabdoid tumors (AT/RT), which are easily ruled out by immunohistochemistry (showing a retained expression of INI1 and BRG1). Contrary to their soft tissue counterparts, the molecular spectrum of CNS tumors (including our data) seems to be wider with different fusion partners for *CIC* and more frequent *ATXN1* fusions [5, 6, 7, 8, 11, 12, 13, 14, 15, 17, 18]. In this study, we reported for the first time a *CIC::EWSR1* fusion. Our study confirmed that *CIC‐*fused and *ATXN1‐*fused sarcomas presented histopathological and epigenetic similarities, clustering together within the same MC [12, 13, 15, 18]. *CIC* or *ATXN1* genes belong to the same complex of transcriptional repressors. ATXN1 is a Chromatin‐binding factor that represses Notch signaling in the absence of Notch intracellular domain by acting as a C‐repeat/DRE binding factor 1 (CBF1) corepressor and in concert with CIC involved in brain development [28]. The current series confirmed that DNA‐methylation proven SARC*‐CIC* may present the *CIC::LEUTX* fusion, which has been recurrently observed in HGNET‐*CIC* [20]. Because HGNET‐*CIC* shares clinical (young age), radiological (presented here in only one tumor) and molecular features (mainly *CIC::LEUTX* fusions, but also a *CIC::NUTM1* fusion in one reported tumor) with SARC*‐CIC*, the question of a same tumor type presenting a wide spectrum of morphology from pure mesenchymal to glioneuronal or subtype may be considered. However, to our knowledge, no SARC‐*CIC* with glial or glioneuronal morphological features has been identified in the literature or in this cohort. The expression of glial and neuronal markers may only represent the pluriphenotypic pattern of these tumors, which is also the case with other CNS tumors, such as AT/RT. According to the original description [20], HGNET‐*CIC* present a different morphology with a predominant glial and/or glioneuronal component (oligodendrocyte‐like features, glial processes, pseudorosettes, eosinophilic granular bodies) and a distinct DNA‐methylation profile [20]. With regards to the literature and the current case, it seems that HGNET‐*CIC* are characterized by a pleomorphism of morphological patterns, similar to what one might observe in pleomorphic xanthoastrocytomas (PXA) or supratentorial ependymomas, *ZFTA*‐fused for example [29]. Like SARC‐*CIC*, we found that the case of HGNET‐*CIC* expressed ETV4 and CD99. In these conditions, ETV4 would be an interesting marker for screening the *CIC* fusions in the spectrum of high‐grade glial/glioneuronal tumors, *IDH*, H3‐ and *BRAF*‐wildtype. In the previous study, Sievers et al. suggested vimentin as a potential differential marker to distinguish sarcomas from HGNET‐*CIC* [20]. However, our experience has shown vimentin to be diffusely expressed by both SARC‐*CIC* and HGNET‐*CIC*. Consequently, the diagnosis of HGNET‐*CIC* remains to be a challenge, and the current epigenetic classifier of brain tumors does not include this MC. This diagnosis may integrate histopathology (glioneuronal tumor) and the presence of a *CIC* fusion. Contrary to SARC*‐CIC*, it seems that HGNET‐*CIC* are only supratentorial [20]. Like their soft tissue counterpart, most SARC‐*CIC* follow an aggressive course with frequent recurrences (61% of patients), most commonly local, and death (45% of patients) [5, 6, 7, 8, 9, 11, 12, 13, 14, 15, 16, 18]. In terms of prognosis, further series of HGNET‐*CIC* are needed to determine their behavior (data are available for only six patients, mean progression‐free survival: 13.5 months and only one patient died 15 months after the initial diagnosis) [20].

In the current WHO Classification, the DNA‐methylation profile constitutes a desirable criterion for the diagnosis of SARC‐*CIC* in the CNS. For laboratories that do not have access to the methylation‐based classification, FISH *CIC* can be used as an important diagnostic tool for this tumor type.

To conclude, SARC‐*CIC* and *HGNET‐*CIC may share similar fusions, clinical and radiological findings, and the same immunoprofile. Further studies are needed for a detailed histopathological characterization of HGNET‐*CIC* to confirm if they represent a novel pleomorphic glial/glioneuronal tumor entity rather than a subtype or variant of SARC‐*CIC*. This work and the recent literature have evidenced that the current terminology for *CIC*‐rearranged sarcoma is too restrictive and the essential criteria should be improved. Because SARC‐*CIC* may present non‐*CIC* fusions, we suggest CNS sarcoma, *CIC/ATXN1* complex‐fused, as a closer consensual nosological term for this group of tumors. In addition, we suggest the following diagnostic criteria: a mesenchymal high‐grade primary CNS tumor having a predominant pattern of small round cell morphology; AND INI1 and BRG1 retained expressions; AND ETV4 expression; AND the presence of a *CIC* or *ATXN1* fusion.

## AUTHOR CONTRIBUTIONS

A.T.E., A.S., E.U.C., F.B., K.M., A.M.E., M.S., C.A.M., B.L., A.E., W.G., T.B., A.K., O.A., J.B., D.C., C.G., and P.V. conducted the histopathological examinations. P.S., D.G., Y.N., A.M., and G.P. conducted the molecular studies. A.T.E., L.H., and P.V. drafted the manuscript; V.D.R., A.G., and N.B. reviewed all imaging data. V.L., G.I., T.B., and K.B. recruited patients, provided samples, and clinical informations. All authors reviewed the manuscript.

## CONFLICT OF INTEREST STATEMENT

The authors declare no conflicts of interest.

## Supporting information


**Supplementary Figure 1.** Treatment and outcome data.

**Supplementary Figure 2.** Dimensionality reduction with uniform manifold approximation and projection (UMAP). Our 14 samples were compared to reference samples (from the DKFZ classifier v12.8 and from the cohort published by Sievers et al., 2023) belonging to CIC‐rearranged sarcoma (CNS_SARC_CIC), Ewing sarcoma (EWS), Diffuse pediatric‐type high grade glioma, MYCN subtype (GBM_pedMYCN), Diffuse paediatric‐type high grade glioma, RTK1 subtype (GBM_pedRTK1), Diffuse paediatric‐type high grade glioma, RTK2 subtype (GBM_pedRTK2), Ganglioglioma (GG), CNS tumor with BCOR internal tandem duplication (HGNET_BCOR), Astroblastoma, MN1‐altered, MN1:BEND2‐fused (HGNET_MN1), Neuroepithelial tumor with PATZ1 fusion (HGNET_PATZ1), High‐grade neuroepithelial tumor, CIC‐fused (HGNET_CIC), Pilocytic astrocytoma, hemispheric (PA_CORT), Pleomorphic xanthoastrocytoma (PXA). Blacks dots represent samples from the current study.

**Supplementary Figure 3.** Copy number variation features.

**Table S1.** Clinical, radiological, histopathological, and molecular data from our cohort.

## Data Availability

The data supporting this study are available from the corresponding author upon reasonable request.
