# Supplementary figures and images for: CIC/ATXN1‐rearranged tumors in the central nervous system are mainly represented by sarcomas: A comprehensive clinicopathological and epigenetic series
Source: Brain Pathol. 2024 Oct 23;35(2):e13303. doi: 10.1111/bpa.13303 (PMC11835441; doi:10.1111/bpa.13303)

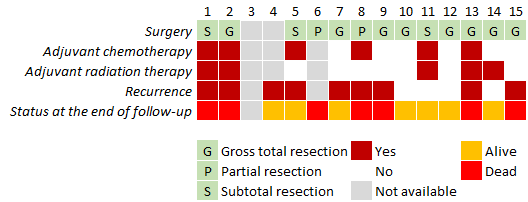

Supplement: Supplementary file 1 — Supplementary Figure 1. Treatment and outcome data. [file BPA-35-e13303-s003.tif]

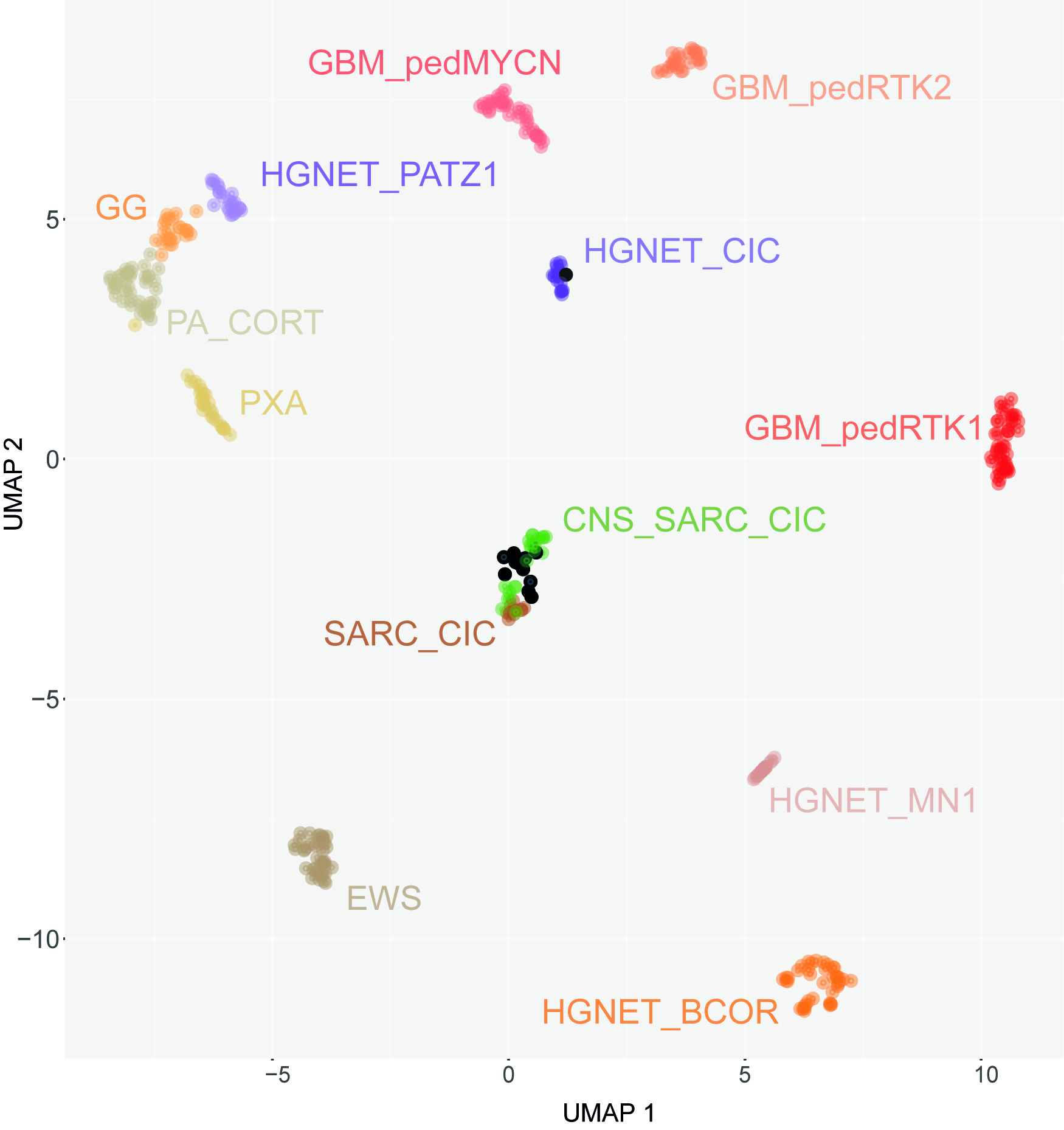

Supplement: Supplementary file 2 — Supplementary Figure 2. Dimensionality reduction with uniform manifold approximation and projection (UMAP). Our 14 samples were compared to reference samples (from the DKFZ classifier v12.8 and from the cohort published by Sievers et al., 2023) belonging to CIC‐rearranged sarcoma (CNS_SARC_CIC), Ewing sarcoma (EWS), Diffuse pediatric‐type high grade glioma, MYCN subtype (GBM_pedMYCN), Diffuse paediatric‐type high grade glioma, RTK1 subtype (GBM_pedRTK1), Diffuse paediatric‐type high grade glioma, RTK2 subtype (GBM_pedRTK2), Ganglioglioma (GG), CNS tumor with BCOR internal tandem duplication (HGNET_BCOR), Astroblastoma, MN1‐altered, MN1:BEND2‐fused (HGNET_MN1), Neuroepithelial tumor with PATZ1 fusion (HGNET_PATZ1), High‐grade neuroepithelial tumor, CIC‐fused (HGNET_CIC), Pilocytic astrocytoma, hemispheric (PA_CORT), Pleomorphic xanthoastrocytoma (PXA). Blacks dots represent samples from the current study. [file BPA-35-e13303-s004.tif]
