# Supplementary material for: CIC/ATXN1‐rearranged tumors in the central nervous system are mainly represented by sarcomas: A comprehensive clinicopathological and epigenetic series
Source: Brain Pathol. 2024 Oct 23;35(2):e13303. doi: 10.1111/bpa.13303 (PMC11835441; doi:10.1111/bpa.13303)

Case 1

Copy number prediction

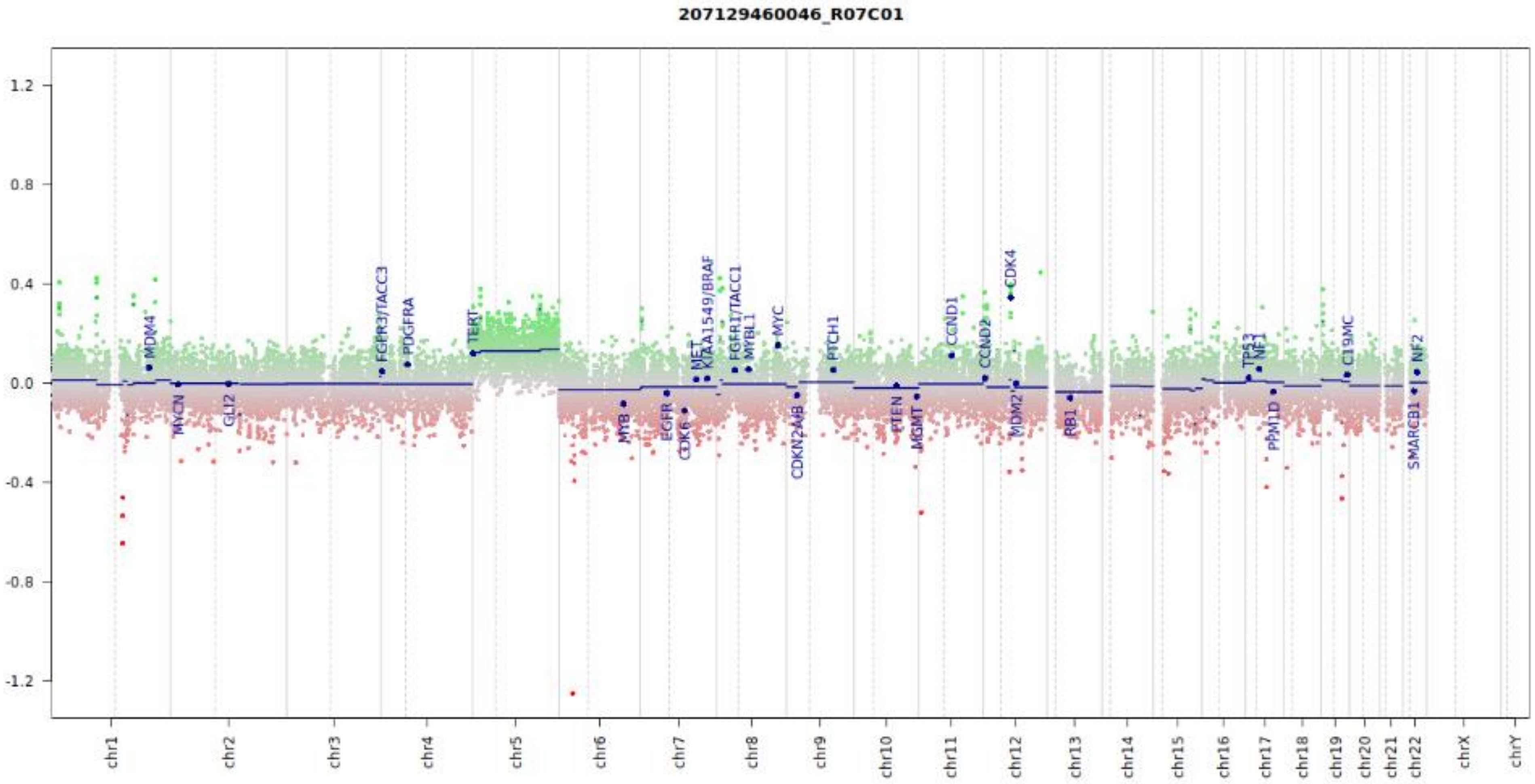

Case 2

Copy number prediction

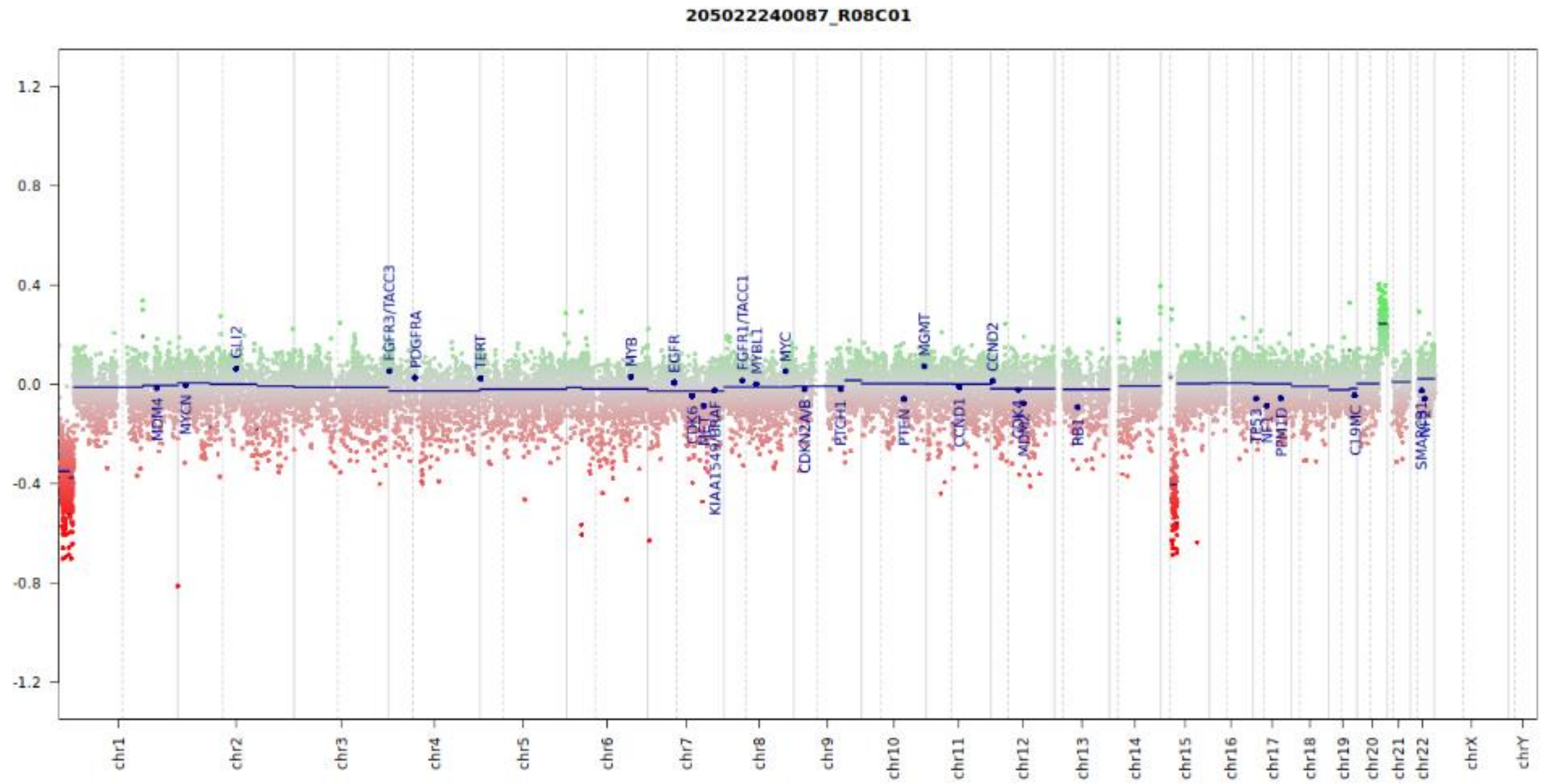

Case 3

Copy number prediction

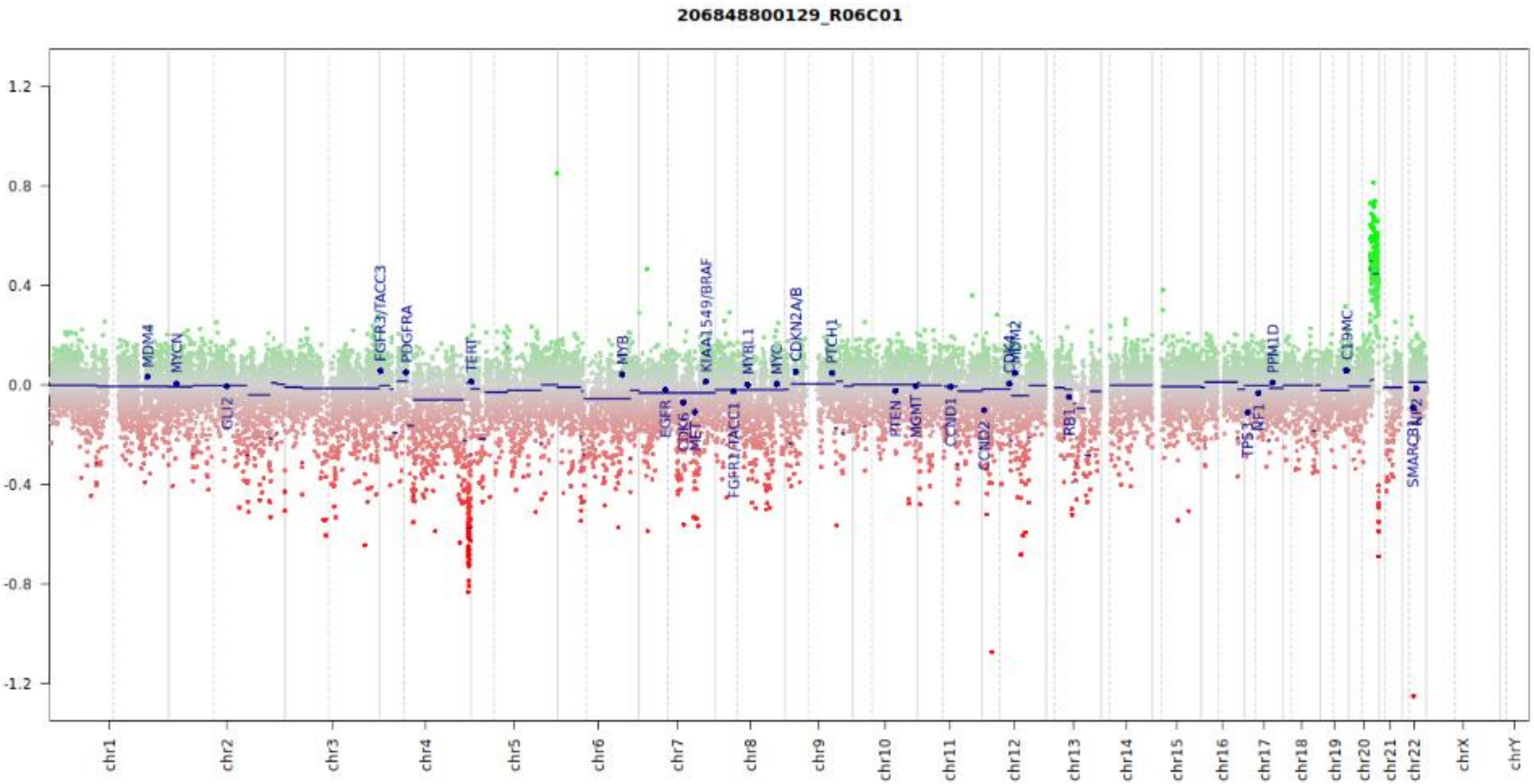

Case 4

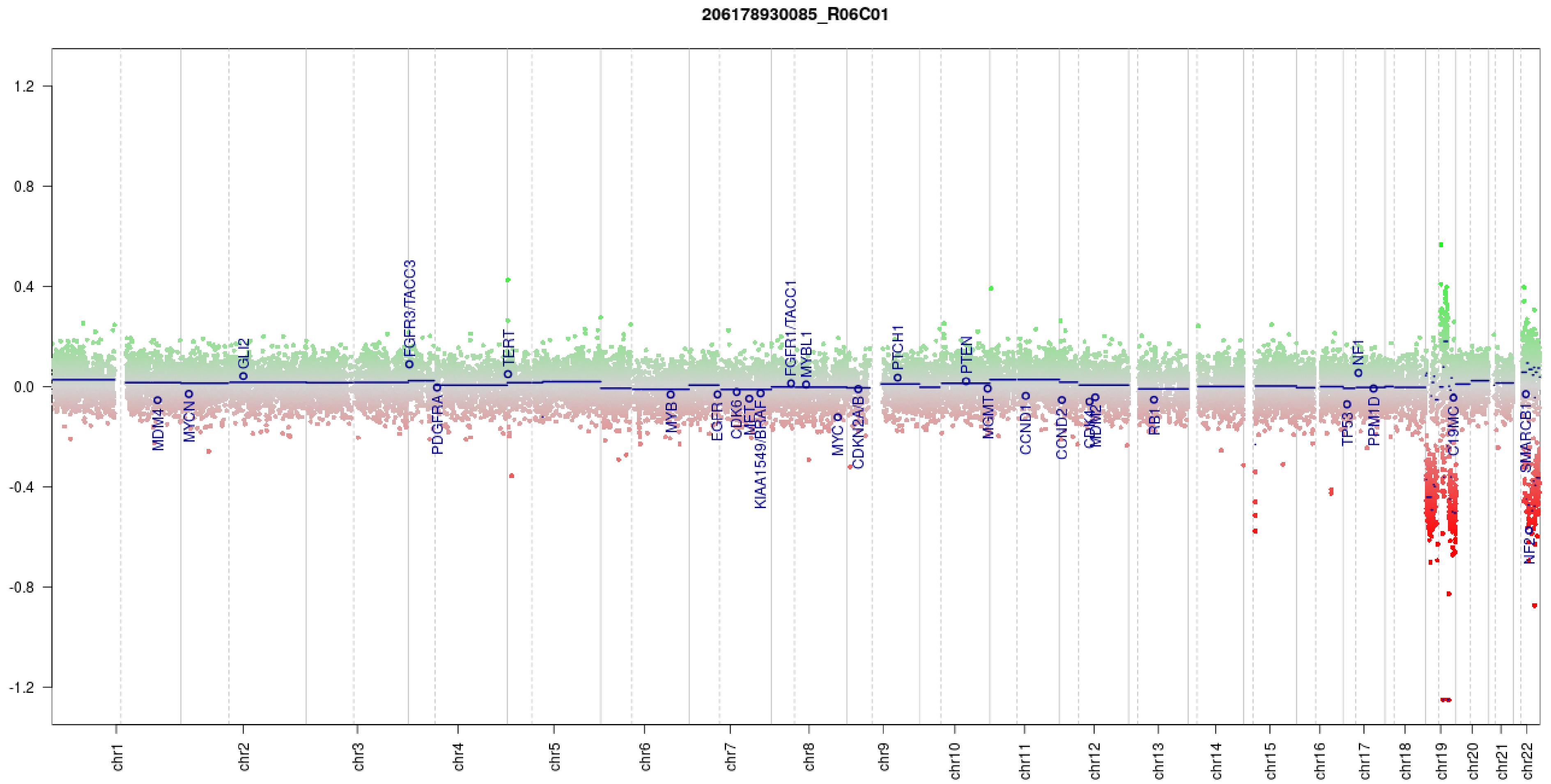

206178930085_R06C01
1.2
0.8
0.4
0.0
-0.4
-0.8
-1.2
MDM4
MYCN
GLI2
FGFR3/TACC3
PDGFRA
TERT
MYB
EGFR
CDK6
MET
KIAA1549/BRAF
FGFR1/TACC1
MYBL1
MYC
CDKN2A/B
PTCH1
PTEN
MGMT
CCND1
CCND2
CDK4
MDM2
RB1
TP53
NF1
PPM1D
C19MC
SMARCB1
NF2
chr1
chr2
chr3
chr4
chr5
chr6
chr7
chr8
chr9
chr10
chr11
chr12
chr13
chr14
chr15
chr16
chr17
chr18
chr19
chr20
chr21
chr22

Case 5

Copy number prediction

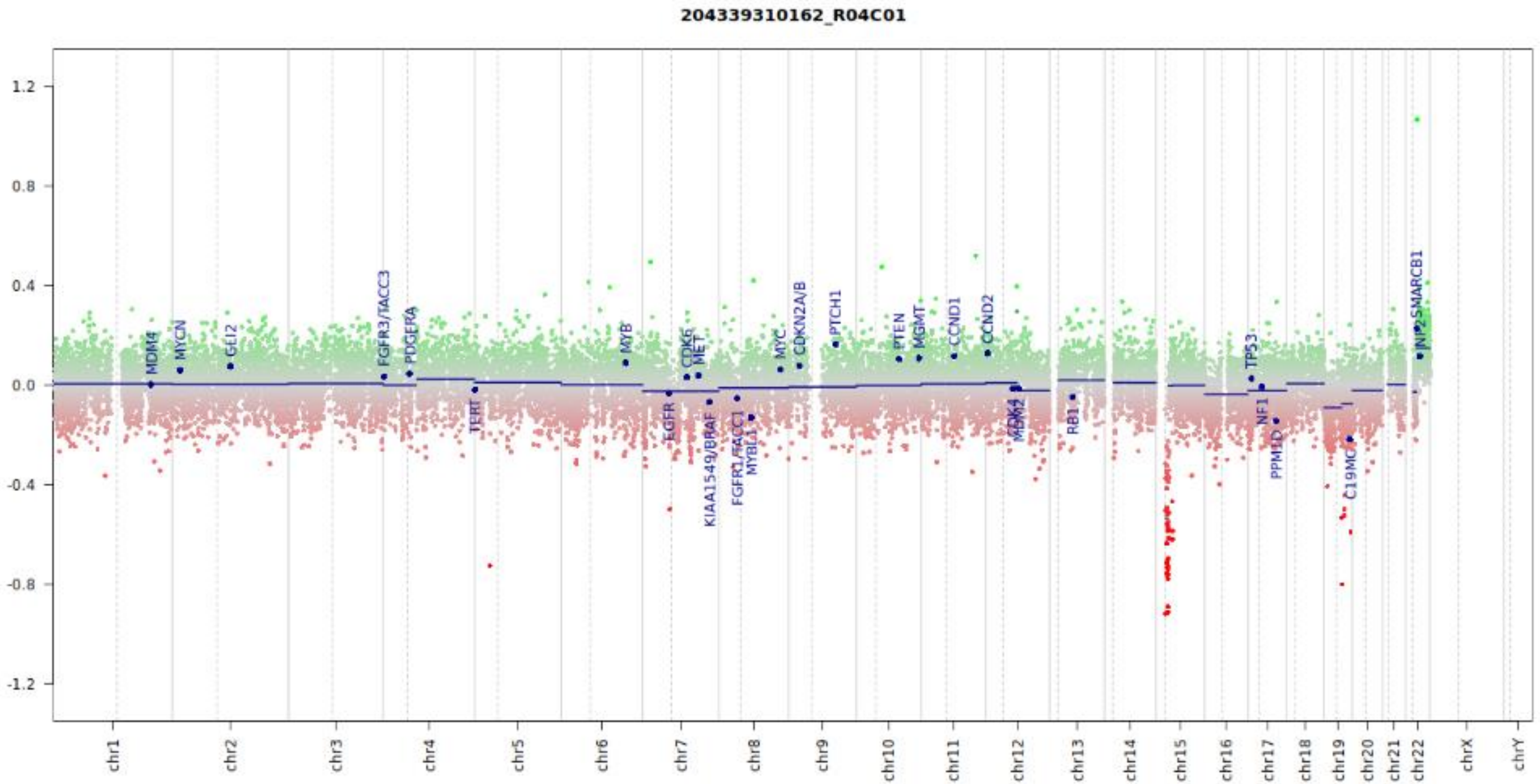

Case 6

Copy number prediction

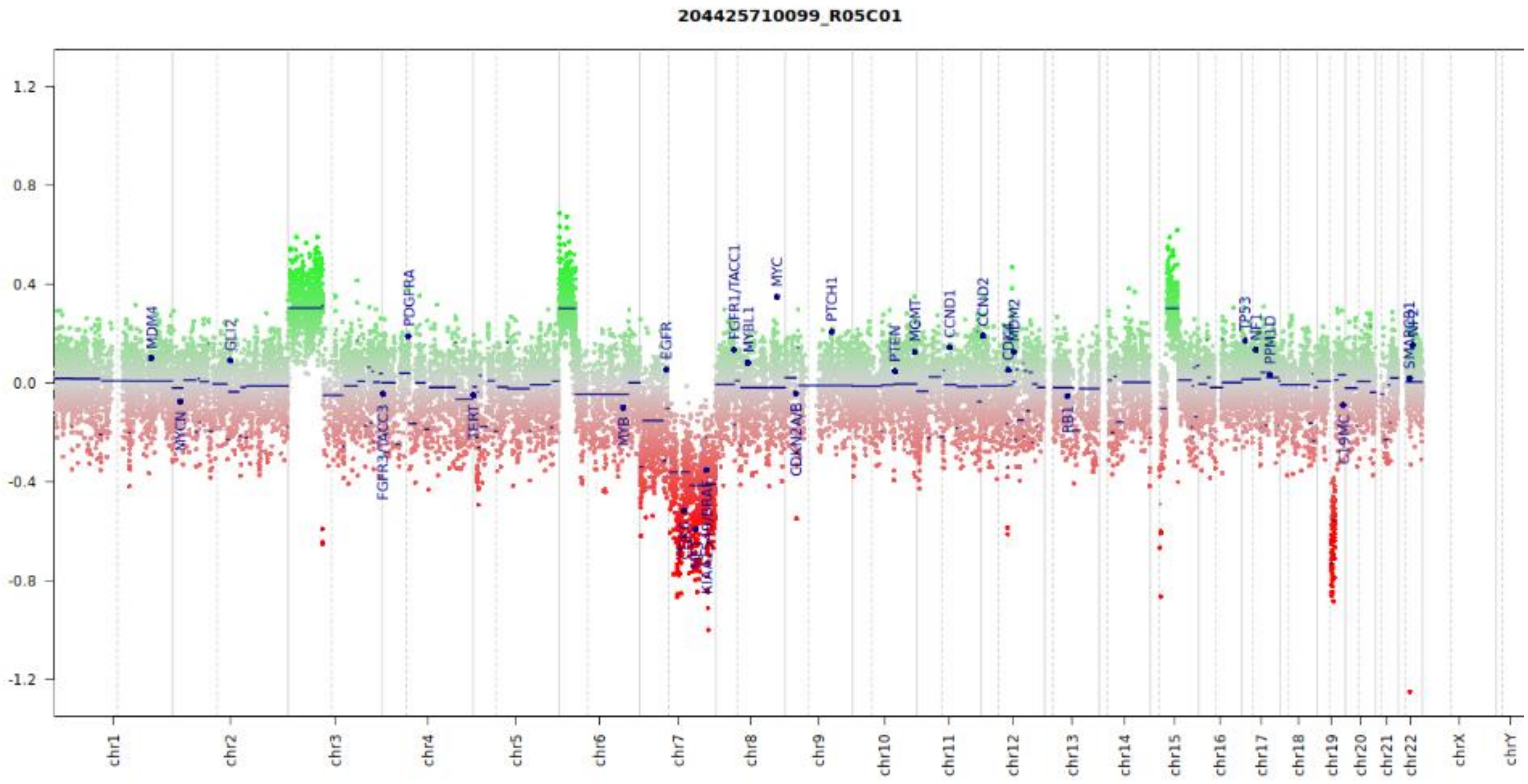

Case 7

Copy number prediction

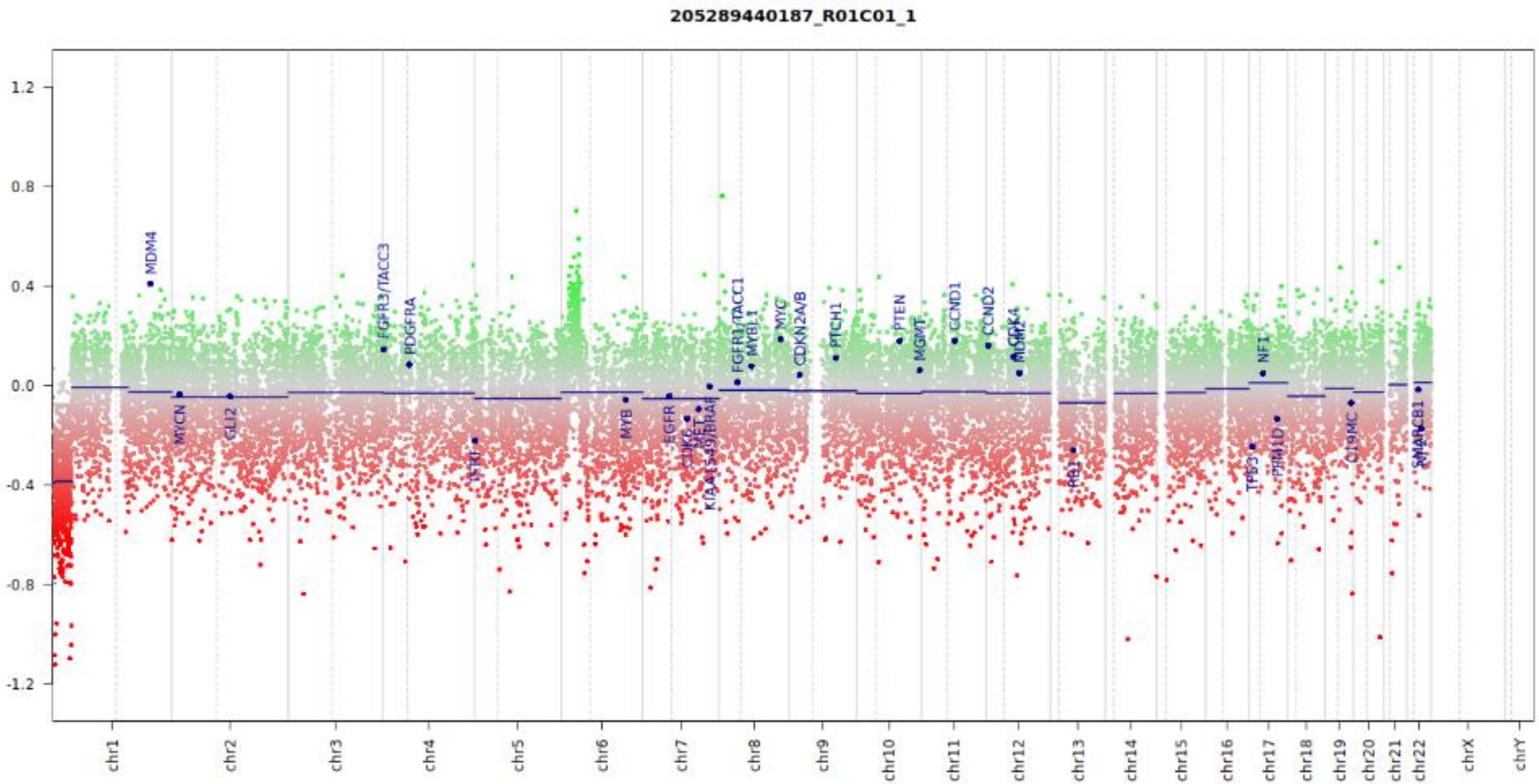

Case 8

Copy number prediction

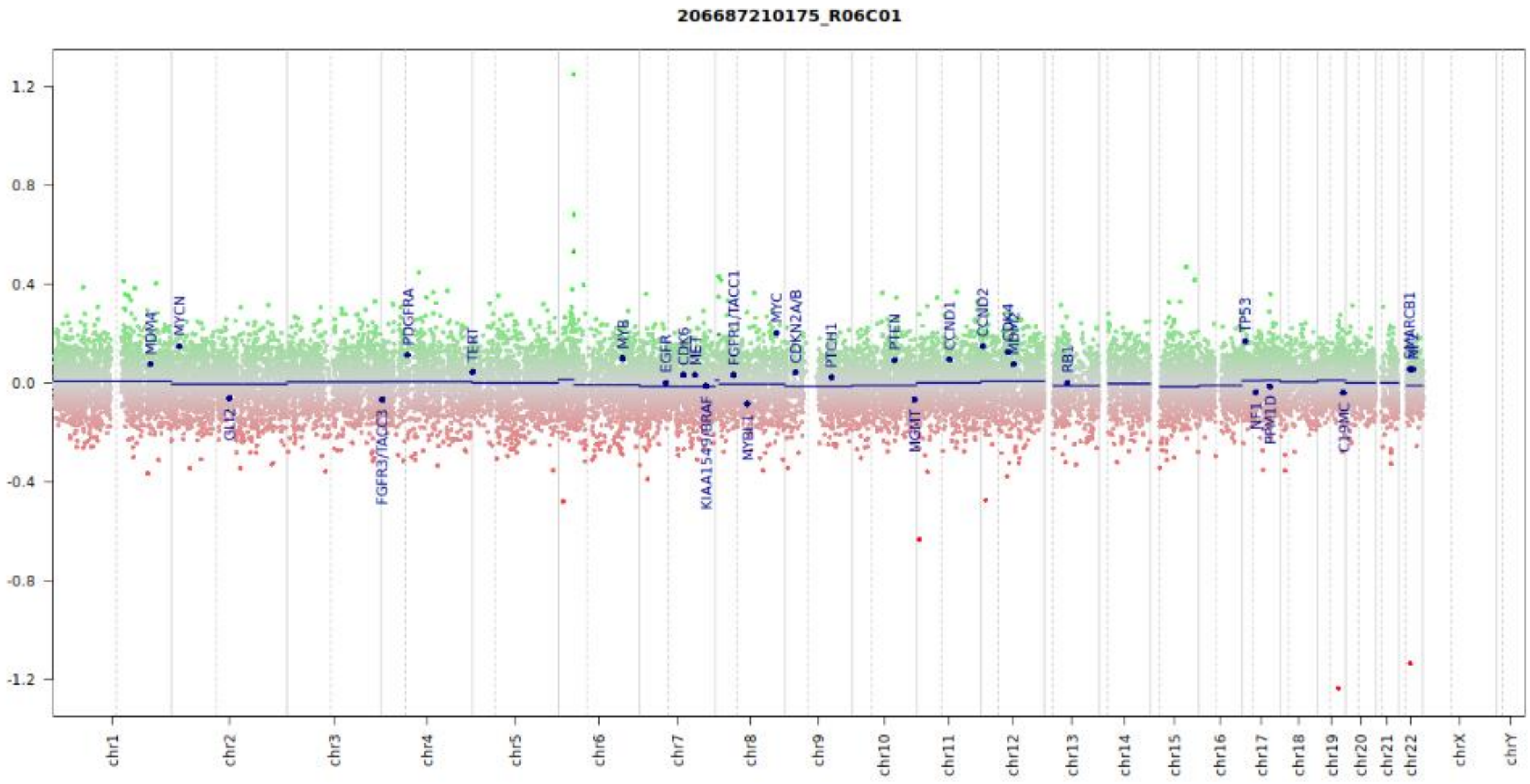

Case 9

Copy number prediction

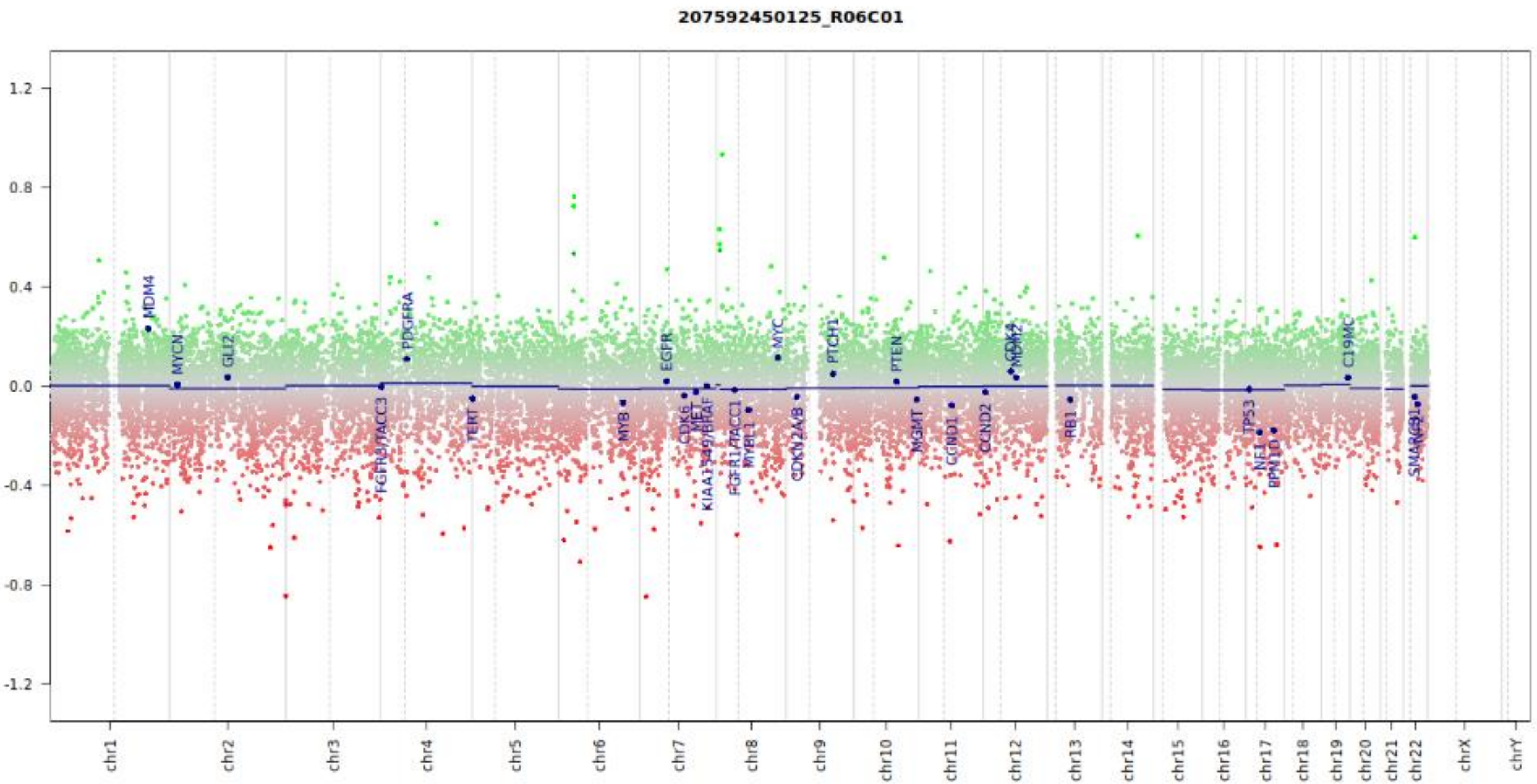

Case 10

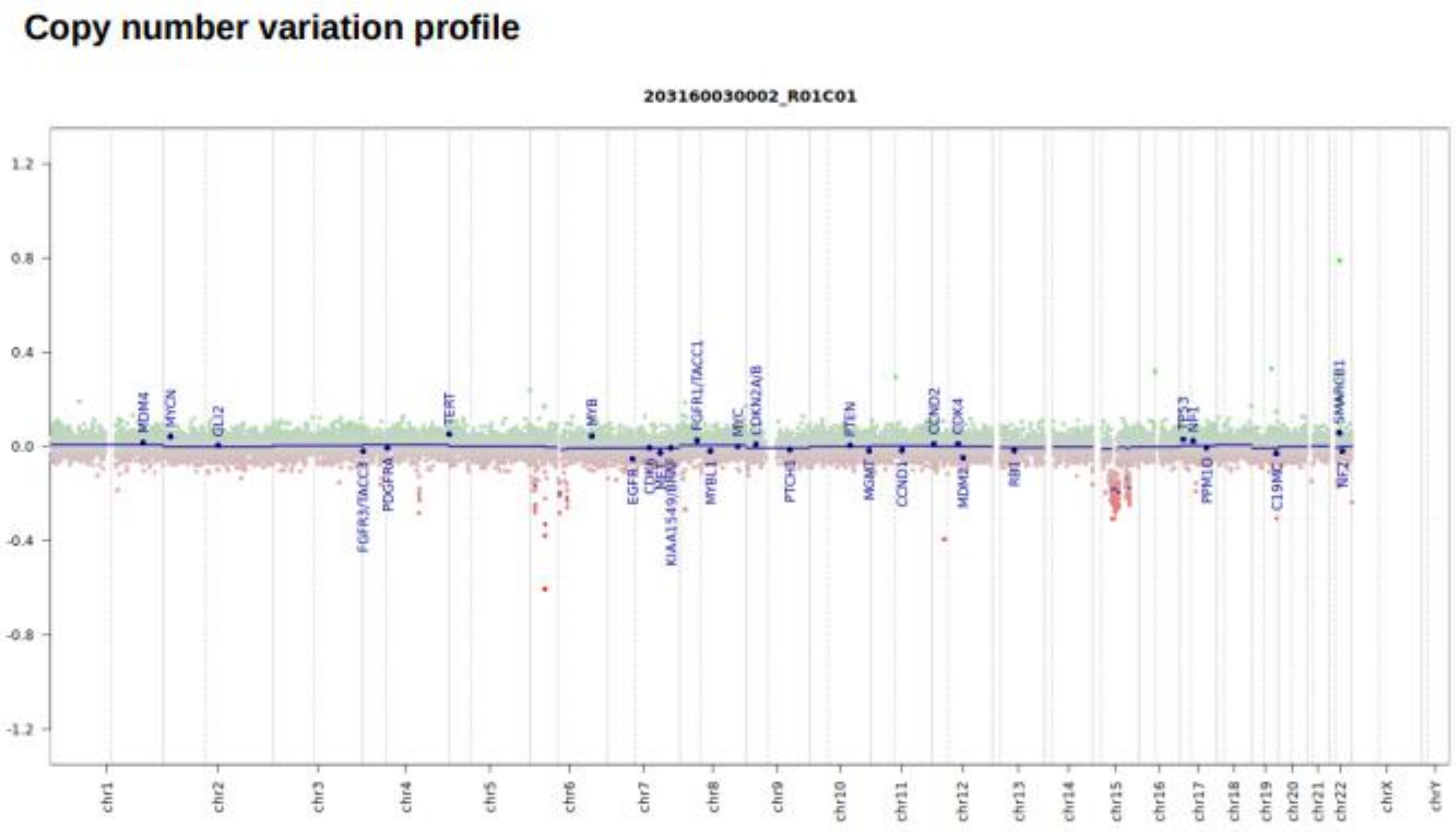
Copy number variation profile
203160030002_R01C01
1.2
0.8
0.4
0.0
-0.4
-0.8
-1.2
MDM4
MYCN
GLI2
FGFR3/TACC3
PDGFRA
TERT
MYB
EGFR
CDK6
MET
FGFR1/TACC1
MYBL1
MYC
CDKN2A/B
PTCH1
PTEN
MGMT
CCND1
CCND2
CDK4
MDM2
RB1
TP53
NF1
PPM1D
C19MC
SMARCB1
NF2
chr1
chr2
chr3
chr4
chr5
chr6
chr7
chr8
chr9
chr10
chr11
chr12
chr13
chr14
chr15
chr16
chr17
chr18
chr19
chr20
chr21
chr22
chrX
chrY

Case 11

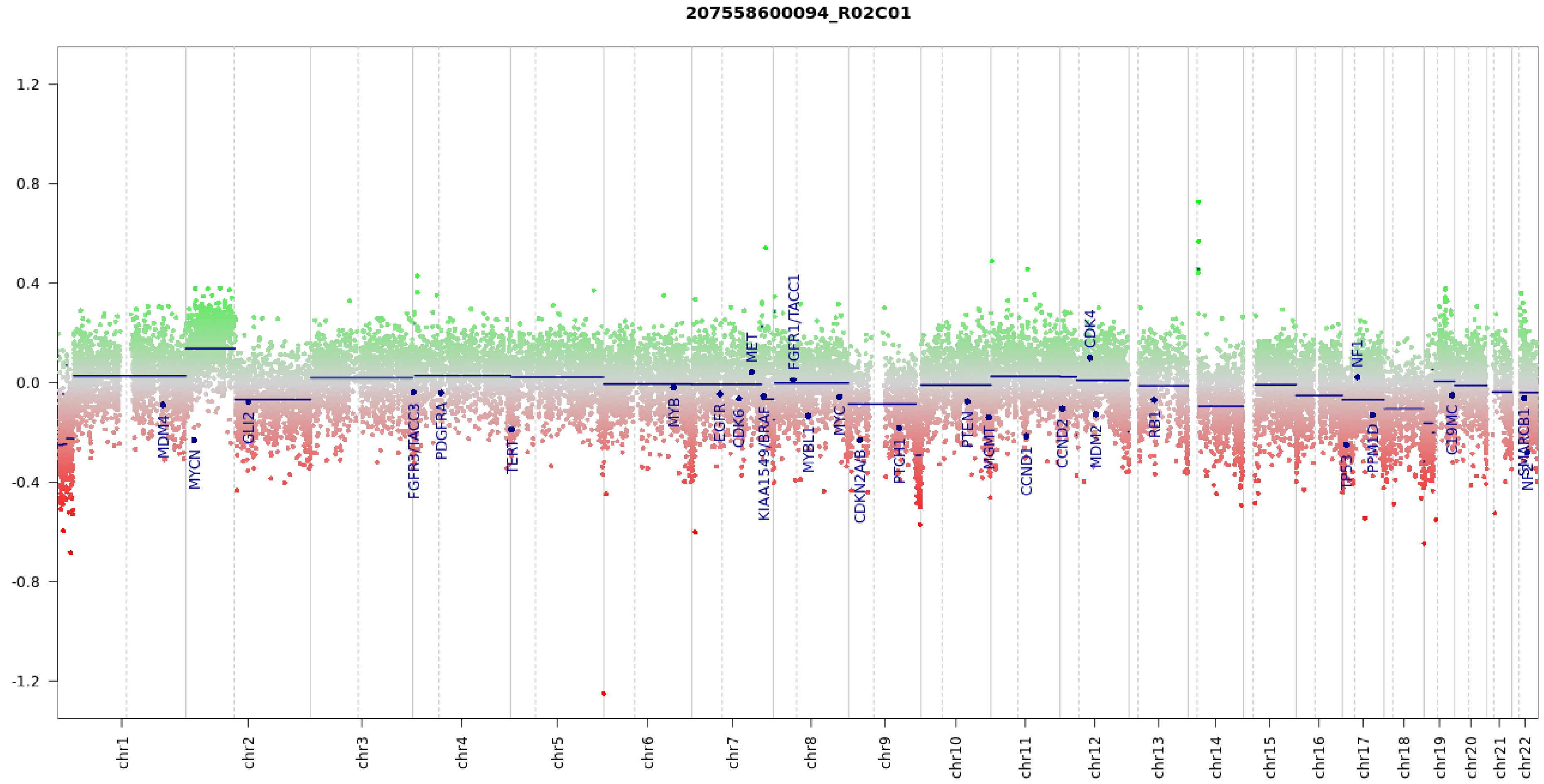
207558600094_R02C01
1.2
0.8
0.4
0.0
-0.4
-0.8
-1.2
chr1
chr2
chr3
chr4
chr5
chr6
chr7
chr8
chr9
chr10
chr11
chr12
chr13
chr14
chr15
chr16
chr17
chr18
chr19
chr20
chr21
chr22
MDM4
MYCN
GLI2
FGFR3/TACC3
PDGFRA
TERT
MYB
EGFR
CDK6
MET
KIAA1549/BRAF
FGFR1/TACC1
MYBL1
MYC
CDKN2A/B
PTCH1
PTEN
MGMT
CCND1
CCND2
CDK4
MDM2
RB1
TP53
NF1
PPM1D
C19MC
SMARCB1
NF2

Case 12

Copy number prediction

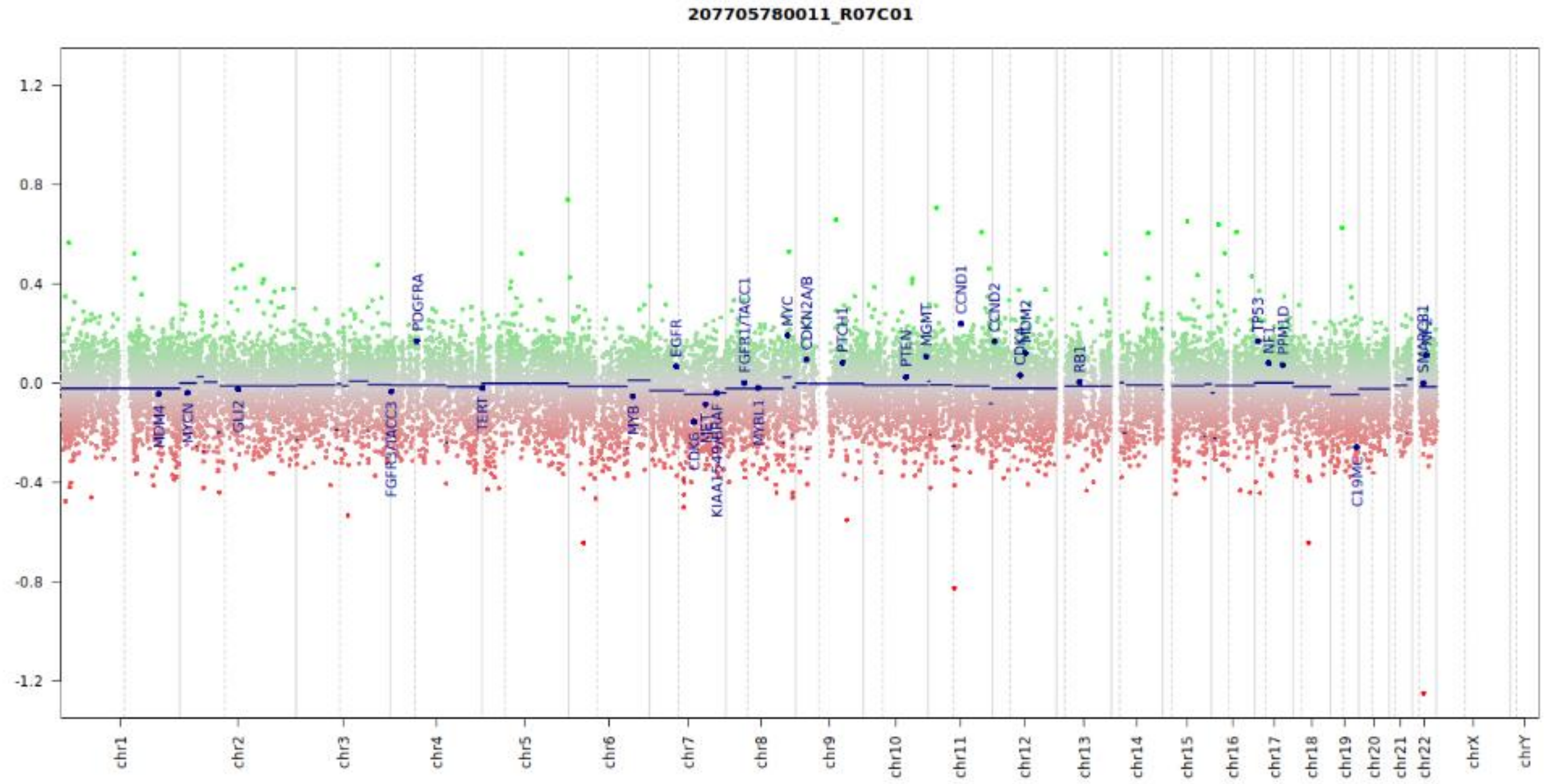

Case 13

Copy number prediction

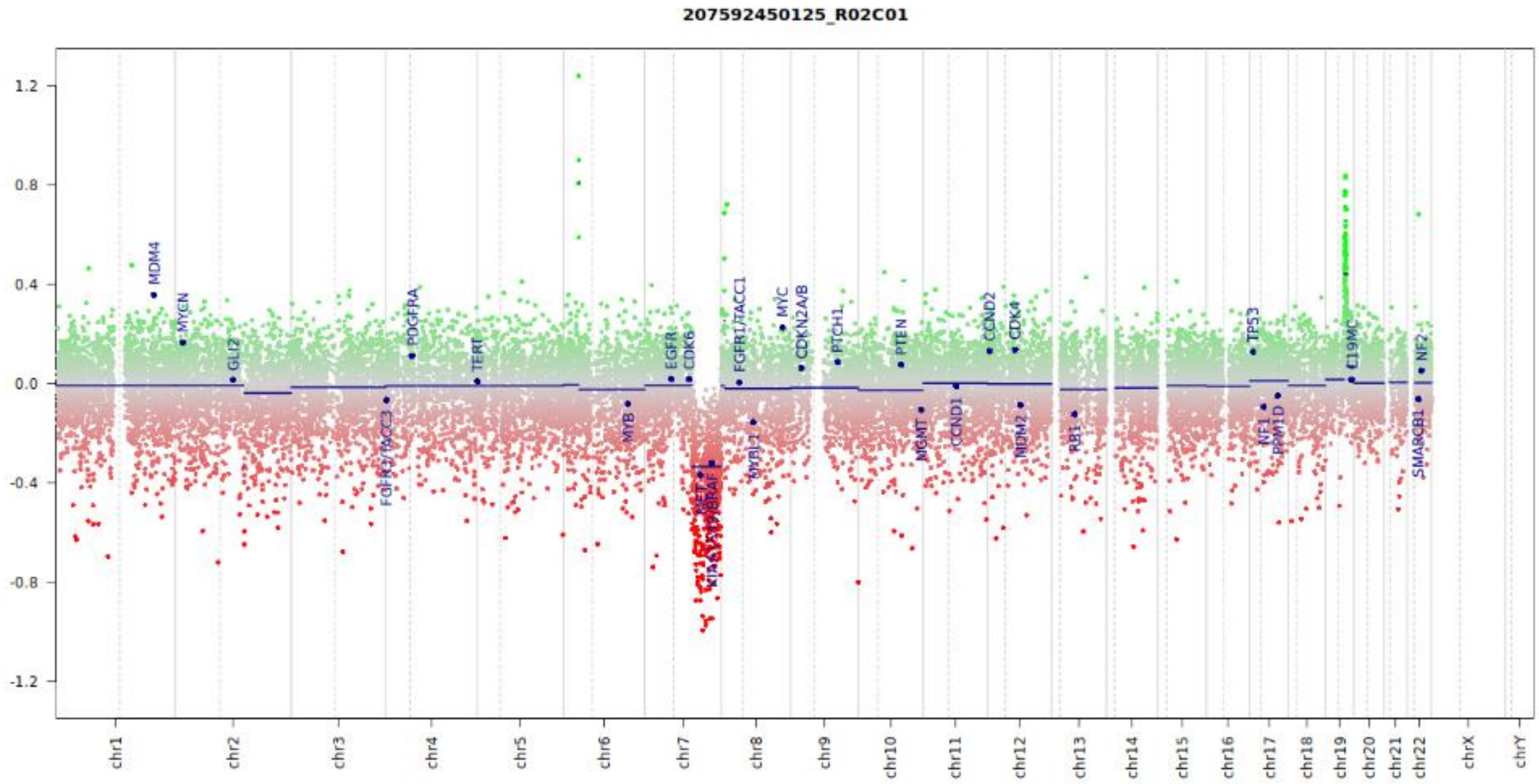

Case 14

Copy number prediction

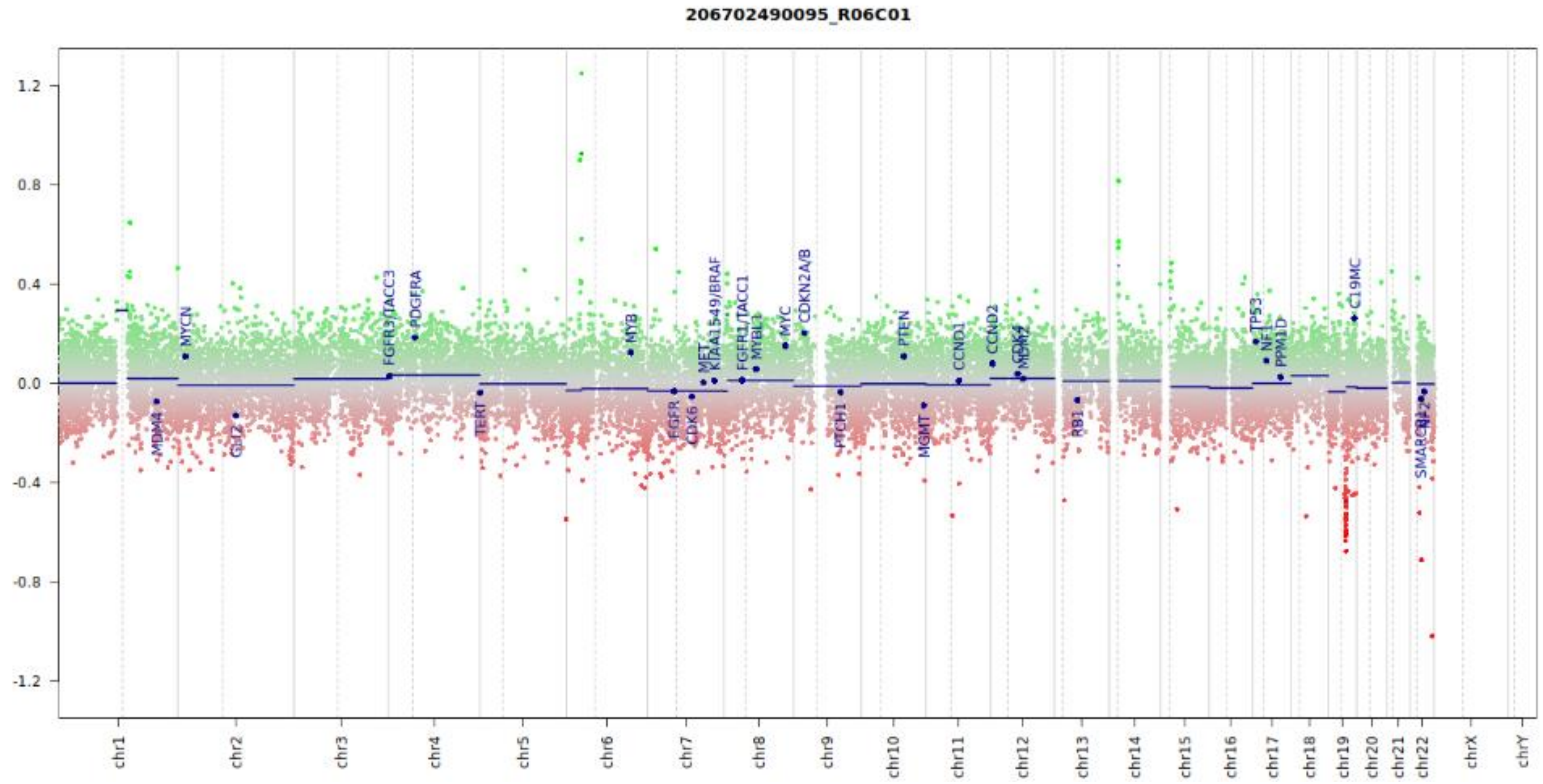

Supplement: Supplementary file 3 — Supplementary Figure 3. Copy number variation features. [file BPA-35-e13303-s002.pdf]
